# Isolation and Identification of Potent Antidiabetic Compounds from *Antrodia cinnamomea*—An Edible Taiwanese Mushroom

**DOI:** 10.3390/molecules23112864

**Published:** 2018-11-02

**Authors:** Hung Tse Huang, San-Lang Wang, Van Bon Nguyen, Yao-Haur Kuo

**Affiliations:** 1 Department of Biochemical Science and Technology, National Taiwan University, Taipei 106, Taiwan; kk49310953@nricm.edu.tw; 2 Division of Chinese Materia Medica Development, National Research Institute of Chinese Medicine, Taipei 11221, Taiwan; 3 Department of Chemistry, Tamkang University, New Taipei City 25137, Taiwan; 4 Life Science Development Center, Tamkang University, New Taipei City 25137, Taiwan; 5 Institute of Research and Development, Duy Tan University, Da Nang 550000, Vietnam; 6 Graduate Institute of Integrated Medicine, College of Chinese Medicine, China Medical University, Taichung 40402, Taiwan; 7 Ph.D. Program for Clinical Drug Development of Chinese Herbal Medicine, College of Pharmacy, Taipei Medical University, Taipei 11031, Taiwan

**Keywords:** *Antrodia cinnamomea*, antidiabetic, edible mushroom, α-glucosidase inhibitor, antcin K, dehydrosulphurenic acid, dehydroeburicoic acid, eburicoic acid

## Abstract

*Antrodia cinnamomea* (AC), an edible Taiwanese mushroom, has been recognized as a valuable natural resource with vast biological and medicinal benefits. Recently, the hypoglycemic and anti-diabetic effects of AC were mentioned in several studies. However, no studies have investigated α-glucosidase inhibitors from AC fruiting bodies (ACFB) as they relate to type 2 diabetes (T2D) treatment. The purpose of this study was to gain evidence of potent α-glucosidase inhibitory effects, as well as isolate, identify and characterize the active compounds of ACFB. The MeOH extract of ACFB demonstrated potent α-glucosidase inhibitory activity, and possessed high pH stability (pH 2–11) and thermostable properties at 40–50 °C. Further purification led to the isolation of eight constituents from ACFB, identified as: 25*S*-antcin K (**1**), 25*R*-antcin K (**2**), dehydrosulphurenic acid (**3**), 25*S*-antcin I (**4**), 25*S*-antcin B (**5**), 25*R*-antcin B (**6**), dehydroeburicoic acid (**7**) and eburicoic acid (**8**). Notably, the ACFB extract and its identified compounds, except **1**, **4**, and **6** demonstrated a greater effect (EC_50_ = 0.025–0.21 mg/mL) than acarbose (EC_50_ = 0.278 mg/mL). As such, these active compounds were determined to be new potent mushroom α-glucosidase inhibitors. These active compounds were also identified on the HPLC fingerprints of ACFB.

## 1. Introduction

The incidence of diabetes mellitus (DM), a chronic metabolic disorder, has been dramatically increasing and reducing people’s quality of life worldwide [1]. People with DM are at high risk for many other complications, including kidney failure, depression, cardiovascular disease, frailty, cognitive decline or premature death [2]. The number of diabetics was reported to be 382 million. Of these, 90% of cases in 2013 were type 2 diabetes (T2D), and the number of total DM cases is estimated to increase to 592 million by 2035 [3]. T2D has been managed by several therapies, including the use of α-glucosidase inhibitors (aGIs) [4]. Currently, several commercial aGIs, such as acarbose, voglibose and miglitol, are available however some side effects have been reported, including diarrhea, flatulence and abdominal discomfort [5]. Therefore, the search for safe and natural sources of active aGIs, along with their isolation and identification, is a high priority.

aGIs may be obtained from several common natural sources, including medicinal herbs, microbial conversion, edible and medicinal mushrooms [5,6,7,8,9,10,11,12,13,14,15,16,17,18,19,20]. Of these, numerous herbal sources show an α-glucosidase inhibitory effect, and their isolated aGI compounds have already been reported [5,6,7,8,9,10]. Similarly, numerous aGI compounds from the culture broth of microbes were recently investigated [1,11,12,13,14,15], and several edible and medicinal mushroom species have been investigated for their enzyme inhibitory effect related to T2D [16,17,18,19].

Edible and medicinal mushrooms were reported to be commonly used as folk medicine in Asian countries to manage various diseases [20,21,22]. Of these, *Antrodia cinnamomea* (AC), an edible Taiwanese mushroom, was recognized early on for its valuable use in Chinese folk medicine to treat diarrhea, hypertension, allergies, abdominal pain, food and drug intoxication, skin itching and tumorigenic diseases [23]. Research shows that AC possesses vast biological activities, including anti-NO, anti-oxidative, anti-metastatic, hepato-protective, anti-hyperlipidemic, immunomodulatory, cardio-protective, neuro-protective, and anticancer activities [24,25,26]. AC also demonstrated a reducing effect on total cholesterol, plasma triglycerides and low-density lipoprotein levels in obese hamsters [27]. Recently, several isolated compounds from AC, including dehydroeburicoic acid [28], ergostatrien-3β-ol [29], antcin K [30] and eburicoic acid [31], showed a hyperglycemic effect and antidiabetic properties via the glucose transporter 4 (GLUT4) and palmitate-treated C2C12 myotubes in mice fed a high-fat diet [31]. Hwang et al. (2015) reported α-glucosidase inhibitory activity in the extracts of *A. cinnamomea* mycelia and concentrated culture filtrate [32]. However, according to our literature review, no studies reported on using α-glucosidase inhibitors from *A. cinnamomea* fruiting bodies for T2D management until now. The object of this study was to establish *A. cinnamomea* as a potent natural source of α-glucosidase inhibitor constituents that could be useful in T2D treatment.

To achieve this goal, *A. cinnamomea* fruiting bodies (ACFB) were extracted by methanol, then evaluated for its α-glucosidase inhibitory activity and stability property. The major active fractions of ACFB were purified for isolation of active compounds by coupling with an α-glucosidase inhibitory assay. Inhibition modes of the inhibitors and the retention times (RT) of these active compounds on the HPLC fingerprint of the ACFB extract were also determined. The results of this study contributed to the catalogue of novel biological activities of AC, as well as its constituents.

## 2. Results and Discussion

### 2.1. New Evidence of A. Cinnamomea as a Potent Natural Source of α-Glucosidase Inhibitors

ACFB were extracted by methanol and used for bioassay. As shown in Figure S1, ACFB demonstrated potent α-glucosidase inhibitory activity with a high level of maximum inhibition at 99% (at 1.2 mg/mL) and a low EC_50_ value of 0.205 mg/mL. Acarbose, a commercial antidiabetic drug, was tested for comparison and showed a lower inhibitory effect (max inhibition = 90.6% at 2.5 mg/mL, EC_50_ = 0.278 mg/mL) than that of ACFB.

Notably, the potent α-glucosidase inhibitory activity of ACFB extract (EC_50_ = 0.205 mg/mL) was a novel finding in this study, and showed higher activity than that of *A. cinnamomea* mycelia extract (EC_50_ = 310 mg/mL), *A. cinnamomea* cultural filtrate extract (EC_50_ = 310 mg/mL) [32] or *Trametes pubescens* fruiting bodies extract (≥1.0 mg/mL) [17]. ACFB also demonstrated comparable or higher activity than other edible mushroom extracts (EC_50_ = 0.0378–0.325 mg/mL) [18,19], culture broths of selected aGI-producing bacterial strains (EC_50_ = 0.038–3.0 mg/mL) [1,11,13,14] and some recently reported herbal extracts (EC_50_ = 0.17–1.42 mg/mL) [6,7,8,9]. The comparison is briefly summarized in Table 1.

### 2.2. Isolation and Identification of Active Constituents from A. cinnamomea

**Figure 1:**
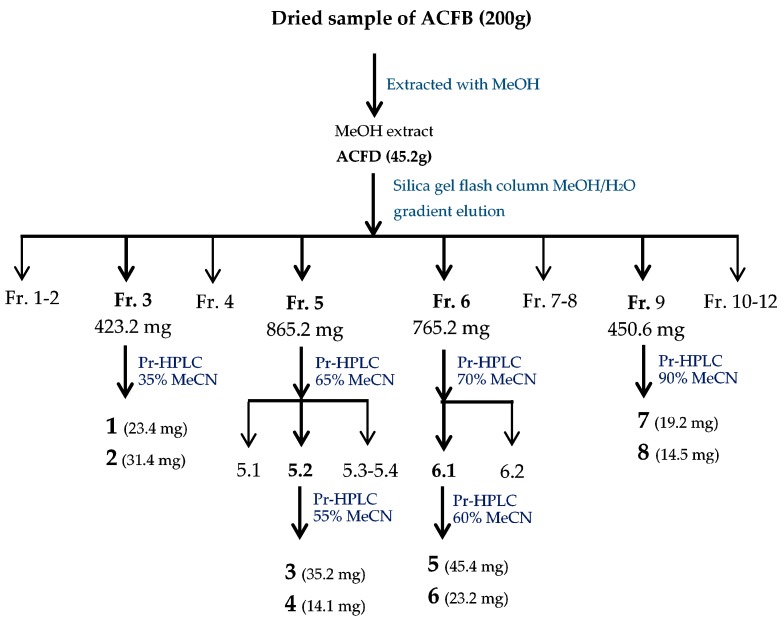
Flow chat of the purification process of active compounds from *Antrodia cinnamomea* fruiting bodies (ACFB) extract.

The methanol extract of ACFB was fractionated and sub-fractionated; active compounds were isolated via silica gel flash column (70–230 mesh, 15 × 10 cm, 0.9 kg) and preparative HPLC (Cosmosil 5C18-AR-II, 5 μm, 250 × 20 mm i.d.). The purification process is briefly summarized in Figure 1.

ACFB extract was primarily separated into 12 fractions via silica column. The four major fractions, ACFB-3, ACFB-5, ACFB-6 and ACFB-9, were eluted with the gradient solvent system of CH_2_Cl_2_/MeOH at a ratio of 17/83–24/76, 33/67–42/58, 43/57–52/48 and 69/31–76-24, respectively. These were then evaluated for aGIs before undergoing further purification. The results in Figure S1a,b in the supplementary section indicate that all four fractions demonstrated potent aGIs with max inhibition and EC_50_ values of 85% and 0.366 mg/mL, 98% and 0.04 mg/mL, 94% and 0.246 mg/mL, and 99% and 0.084 mg/mL, respectively. Of these, fractions ACFB-5 and ACFB-9 possessed the strongest activity due to their small EC_50_ values, ranked at *F* level based on Duncan’s multiple range test at α = 0.01. The other two fractions, ACFB-3 and ACFB-6, showed acceptable activity compared to the crude extract and positive control (acarbose). Further sub-separation and recycling via preparative HPLC resulted in eight compounds.

All purified compounds were evaluated by bioassay primarily for their aGI activity at a concentration of 0.25 mg/mL; the results are presented in Figure S1c. Compounds **2**, **3**, **7** and **8** demonstrated good activity (89–100%), while compounds **1** and **5** possessed activity in the range of 37–60.5%, which were comparable to that of acarbose (44%). Compounds **4** and **6** showed no significant effect against α-glucosidase (≤4%).

The eight isolated compounds **1**–**8** were identified as 25*S*-antcin K (**1**) [33], 25*R*-antcin K (**2**) [33], dehydrosulphurenic acid (**3**) [34], 25*S*-antcin I (**4**) [35], 25*S*-antcin B (**5**) [33], 25*R*-antcin B (**6**) [35] dehydroeburicoic acid (**7**) [36] and eburicoic acid (**8**) [36] by analyzing NMR data, mass spectrometry and comparison to reported compounds. The ^13^C-NMR, ^1^H-NMR data are recorded in the supplementary section as Tables S1 and S2, respectively. The ^13^C-NMR, ^1^H-NMR and mass spectra of all eight identified compounds are also presented in the supplementary section as Figures S3–S26, while their chemical structures are presented in Figure 2.

Recently, compounds **1** and **2** were isolated from ACFB [30,37,38,39] and investigated for its reducing effects on blood glucose, total cholesterol, triglyceride and leptin levels in mice fed a high-fat diet (HFD) [30], as well as its antiproliferative activity [33,37] and inhibition against DEN-enhanced hepatocellular inflammation, fibrosis and carcinoma [38]. Compounds **3**, **7** and **8** were also obtained from ACFB [31,38,39,40].

Compounds **1**, **2** [30], **7** [28], and **8** [31] also showed hyperglycemic and antidiabetic properties via the glucose transporter 4 (GLUT4) and palmitate-treated C2C12 myotubes in mice fed a high-fat diet. Recently, compound **8** was investigated for its anti-type 1 diabetes and hypolipidemic activities [40]. However, potent aGI activity related to type 2 diabetes or obesity treatments had not been reported in the literature for all six active compounds. As such, they were determined to be new mushroom aGIs. The results of this study contributed to the catalogue of novel biological activities of *A. cinnamomea*, as well as its constituents.

### 2.3. Identification of Active Compounds on ACFB Extract Fingerprints

**Figure 3:**
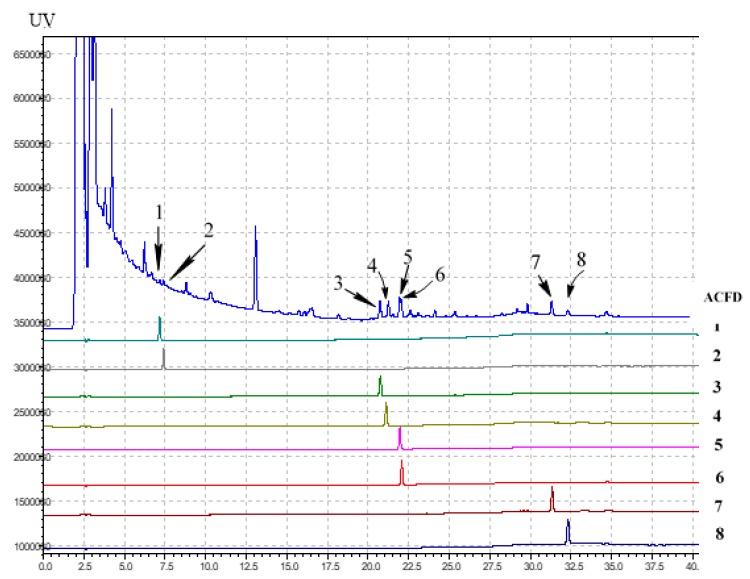
Identification of active inhibitors on the HPLC fingerprints of ACFB extract. 25*S*-antcin K (**1**), 25*R*-antcin K (**2**), dehydrosulphurenic acid (**3**), 25*S*-antcin I (**4**), 25*S*-antcin B (**5**), 25*R*-antcin B (**6**), dehydroeburicoic acid (**7**) and eburicoic acid (**8**). Analysis conditions: The mobile phase consisted of water containing 0.1% phosphoric acid and acetonitrile (ACN) using a gradient program of 40–50% ACN from 0−12 min, 50−60% ACN from 12−17 min, 60−95% ACN from 17−26 min and 95−100% ACN from 26−50 min; separation column (Cosmosil 5C_18_-AR-II, 5 μm, 250 × 4.6 mm i.d.) was employed, eluting at a flow rate of 1.0 mL/min at 35 °C; the real-time UV absorption was detected at 210 nm.

To identify the active inhibitor compounds on HPLC fingerprints of ACFB extract, the crude extract, as well as all potent inhibitor compounds, were analyzed via Cosmosil 5C_18_-AR-II column under the same conditions. The eight isolated compounds were successfully recognized in the HPLC fingerprints of ACFB extract at the retention times (RT) of 7.282 min (**1**), 7.543 min (**2**), 20.896 min (**3**), 21.122 min (**4**) 22.069 min (**5**), 22.084 min (**6**) 31.425 min (**7**), and 32.467 min (**8**) (Figure 3).

Almost all of the active inhibitors could be clearly observed in the HPLC fingerprints of the crude extract. These results indicate that they should be major constituents of ACFB extract. As such, HPLC analysis coupled with inhibitory assay may be a convenient, rapid and precise method to standardize ACFB extracts from different sources in order to select good material for T2D management.

### 2.4. Inhibitory Activity Comparison of α-Glucosidase Inhibitor Compounds

To evaluate the most potent aGI compounds, all identified compounds and acarbose were tested for activity over a large concentration range of 0.02–0.25 μg/mL. Activity was expressed as EC_50_ (mg/mL) and inhibition (%), as presented in Table 2. Of the tested inhibitors, three compounds, dehydrosulphurenic acid (**3**), dehydroeburicoic acid (**7**) and eburicoic acid (**8**), demonstrated the greatest effect against α-glucosidase due to their small EC_50_ values (0.012–0.05 mg/mL, ranked at *d* level), and greatest max inhibition at 0.25 mg/mL (98–100%, ranked at *a* level). The compounds 25*R*-antcin K (**2**) and 25*S*-antcin B (**5**) were also potent inhibitors since their activity was stronger than that of the control, while compound **1** showed the weakest activity.

The potent inhibitory activities of the tested inhibitors, in order of decreasing activity, are as follows: Eburicoic acid (**8**) ≥ dehydroeburicoic acid (**7**) ≥ dehydrosulphurenic acid (**3**) ≥ 25*R*-antcin K (**2**) ≥ 25*S*-antcin B (**5**) ≥ acarbose ≥ 25*S*-antcin K (**1**), where “≥” indicates that there is no significantly higher inhibitory activity between the two inhibitors, based on Duncan’s multiple range test. The results indicate that almost all inhibitors isolated from ACFB extract showed higher activity than acarbose. As such, this edible medicinal mushroom may have potential as a safe and natural source of aGIs for effective management of T2D.

### 2.5. pH and Thermal Stabilities of A. cinnamomea a-Glucosidase Inhibitors

**Figure 4:**
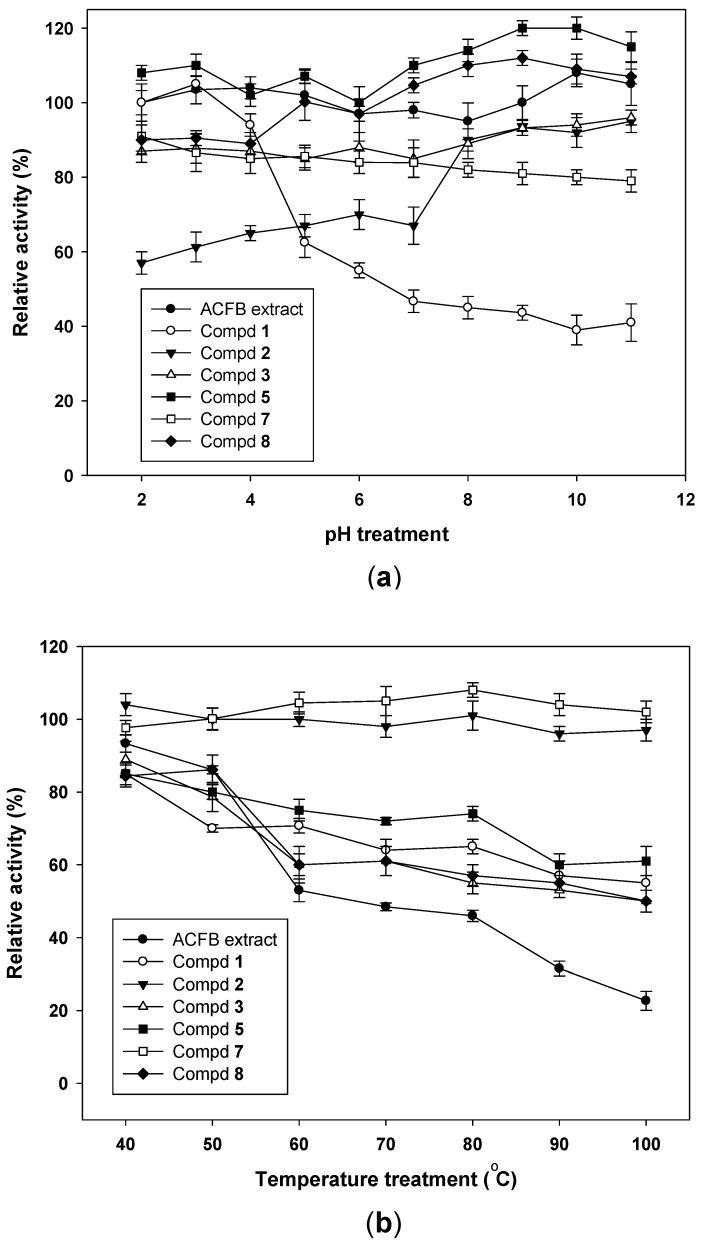
The pH and thermal stabilities of ACFB extract and the purified compounds. The pH (**a**) and thermal (**b**) stability of ACFB and its purified compounds were tested by treating the samples with a range of pH (2–11) and temperatures (40–100 °C) for 30 min, respectively. The α-glucosidase inhibition of treated samples was tested under the same conditions, using the bioassay mentioned in the methods section. Tests were performed in triplicate. Results are means ± SD.

To determine pH stability of *A. cinnamomea* aGIs, each sample was pre-treated with a large pH range of 2–11 for 30 min before evaluating inhibition at pH 7, using the bioassay techniques described in the methods section. As shown in Figure 4a, ACFB extract and the purified compounds (**3**, **5**, **7**, and **8**) possessed high pH stability with great relative activity of 80–117%. Compound **1** demonstrated high stability in acidic pH treatment (pH 2–4) but very weak activity in the pH range treatment from 5–11, while compound **2** showed good activity in the alkaline pH but weak activity in acidic condition treatment. It was suggested that pH stability is an important characteristic that should be considered when evaluating aGIs. A potential aGI should have acceptable or high pH stability, especially at acidic pH, since the pH in the gastrointestinal tract (stomach) is normally acidic [1,5,14]. Recently, the acidic pH stability of some inhibitors was reported, such as aGIs from *Euonymus laxiflorus* Champ which showed low relative activity of 48% at pH 4 and aGIs from *Dalbergia tonkinensis* which demonstrated an acceptable relative activity of 80–83% at pH 2–4, while fermented nutrient broth and fermented squid pens by *Paenibacillus* sp. had potent relative activities of 86–97% (pH 1–4) and 89–95% (pH 2–4), respectively. In this study, ACFB extract and its purified compounds, except compound **2**, demonstrated comparable or higher stability than those of previous reports with a relative activity of 85–110% at an acidic pH of 2–4.

The thermal stability of aGIs from ACFB was evaluated by pre-treating samples to a range of temperatures (40–100 °C) for 30 min, before inhibition was tested at 37 °C by bioassay. The results are presented in Figure 4b. Compounds **2** and **7** showed good thermal stability at all the treated temperatures with high relative activity of 96–108 °C. However, ACFB extract and other identified compounds were only stable at 40–50 °C with relative activity of 70.2–93.3%. The results suggest that ACFB should be not treated higher than 50 °C to obtain aGIs for use in folk medicine or to prepare the extract for purification, since it may reduce significant inhibitory activity. In comparison, aGIs from ACFB except **2** and **7** demonstrated lower thermal stability (22.7–93.3%) than those of aGIs from *E. laxiflorus* Champ. (75–100%) [5], fermented nutrient broth (92–99%) [14] or squid pens fermented by *Paenibacillus* sp. (91–99%) [1].

## 3. Materials and Methods

### 3.1. Materials

The fruiting bodies of *A. cinnamomea* were collected from the mountains of Kavulungan, Taitung, Taiwan in March 2017. *Saccharomyces cerevisiae* α-glucosidase and acarbose were purchased from Sigma Chemical Co., St. Louis City, MO, USA. The substrate *p*NPG was purchased from Sigma Aldrich, St. Louis, MO, USA. Solvents, reagents and other chemicals were obtained at the highest grade available

### 3.2. Determination of α-Glucosidase Inhibitory Activity

The α-glucosidase inhibition was performed following the assay methods described by Nguyen et al. (2018) [15], with slight modifications. In brief, 50 μL sample solutions were mixed with 100 μL α-glucosidase, then incubated at 37 °C for 20 min. A total of 50 μL of *p*-NPG (10 mmol/L) was added to each mixture to start a reaction. The mixture was kept at 37 °C for 30 min before 100 μL Na_2_CO_3_ (1 mol/L) was added to stop the reaction. The final mixture was then measured at 410 nm (A). The control group was also prepared as described above but with the use of 50 μL, 0.1 mol/L potassium phosphate buffer (pH 7) instead of the sample solution; its absorbance was also recorded at 410 nm (B). The α-glucosidase inhibition (%) was estimated using the following equation:

α-glucosidase inhibition (%) = (B − A)/B × 100

Inhibition activity was also expressed as an EC_50_ value, which is defined as the concentration of inhibitor that inhibits 50% of enzyme activity [15]. The crude extract, fractions and pure compounds were prepared in methanol then diluted in 0.1 mol/L potassium phosphate buffer (pH 7). The enzyme solution was also prepared in 0.1 mol/L potassium phosphate buffer (pH 7). Acarbose was prepared in distilled water then also diluted in the same buffer.

### 3.3. Extraction and Purification

General experimental procedures: High resolution electronic ionization mass spectrometry (HREIMS) data were measured using a Shimadzu IT-TOF HR mass spectrometer (Shimadzu, Kyoto, Japan). Nuclear magnetic resonance (NMR) spectra were recorded on a Bruker AC-400 FT-NMR (Bruker BioSpin, Rheinstetten, Germany) using C_5_D_5_N (pyridine-*d*_5_) as solvent. Silica gel 60 (Merck 70−230 and 230−400 mesh, Merck, Darmstadt, Germany) were used for column chromatography, and pre-coated silica gel (Merck 60 F-254) plates were used for TLC. The spots on TLC were detected by spraying with an anisaldehyde-sulfuric acid solution and then heating at 100 °C. HPLC separations were performed on a Shimadzu LC-2040C series apparatus (Shimadzu, Kyoto, Japan) with a photodiode array detector, equipped with a 250 × 4.6 mm i.d. preparative Cosmosil 5C_18_ AR-II column (Nacalai Tesque, Inc., Kyoto, Japan).

Extraction and isolation: Dried *A. cinnamomea* fruiting bodies (200 g) were extracted five times with MeOH (10 L) at 50 °C for 12 h, then concentrated under reduced pressure. The MeOH extract (45.2 g) was separated by silica gel flash column (70–230 mesh, 15 × 10 cm, 0.9 kg) with a gradient solvent system of CH_2_Cl_2_ 100% to MeOH 100%, to provide 12 fractions (ACFB.1–ACFB.12). ACFB.3 (423.2 mg) was purified by preparative HPLC (Cosmosil 5C_18_-AR-II, 5 μm, 250 × 20 mm i.d., MeCN/H_2_O containing 0.1% formic acid, 35:65, flow rate 10 mL/min) to produce 25*S*-antcin K (**1**, 23.4 mg, *^t^*_R_ = 7.282 min) and 25*R*-antcin K (**2**, 31.4 mg, *^t^*_R_ = 7.543 min). ACFB.5 (865.2 mg) was purified by preparative HPLC (Cosmosil 5C_18_-AR-II, 5 μm, 250 × 20 mm i.d., MeCN/H2O containing 0.1% formic acid, 65:35, flow rate 10 mL/min) to produce four subfractions (ACFB.5.1–ACFB.5.4). ACFB.5.2 (232.1 mg) was further purified by recycled preparative HPLC (Cosmosil 5C_18_-AR-II, 5 μm, 250 × 20 mm i.d., MeCN/H2O containing 0.1% formic acid, 55:45, flow rate 10 mL/min, recycled 8 times) to produce dehydrosulphurenic acid (**3**, 35.2 mg, *^t^*_R_ = 20.896 min) and 25*S*-antcin I (**4**, 14.1 mg, *^t^*_R_ = 21.122 min). ACFB.6 (765.2 mg) was purified by preparative HPLC (Cosmosil 5C_18_-AR-II, 5 μm, 250 × 20 mm i.d., MeCN/H_2_O containing 0.1% formic acid, 70:30, flow rate 10 mL/min) to produce two subfractions: ACFB.6.1 and ACFB.6.2. ACFB.6.1 was further purified by recycled preparative HPLC (Cosmosil 5C_18_-AR-II, 5 μm, 250 × 20 mm i.d., MeCN/H_2_O containing 0.1% formic acid, 60:40, flow rate 10 mL/min, recycled 6 times) to produce 25*S*-antcin B (**5**, 45.4 mg, *^t^*_R_ = 22.069 min) and 25*R*-antcin B (**6**, 23.2 mg, *^t^*_R_ = 22.084 min). ACFB.9 (450.6 mg) was purified by preparative HPLC (Cosmosil 5C_18_-AR-II, 5 μm, 250 × 20 mm i.d., MeCN/H_2_O containing 0.1% formic acid, 90:10, flow rate 10 mL/min) to produce dehydroeburicoic acid (**7**, 19.2 mg, *^t^*_R_ = 31.425 min) and eburicoic acid (**8**, 14.5 mg, *^t^*_R_ = 32.467 min).

### 3.4. HPLC Analysis

Separation column (Cosmosil 5C_18_-AR-II, 5 μm, 250 × 4.6 mm i.d.) was used, while eluting at a flow rate of 1.0 mL/min at 35 °C. The mobile phase consisted of water containing 0.1% phosphoric acid and acetonitrile (ACN), using a gradient program of 40−50% ACN from 0−12 min, 50−60% ACN from 12−17 min, 60−95% ACN from 17−26 min and 95−100% ACN from 26−50 min. The real-time UV absorption was detected at 210 nm. Each isolated compound was accurately weighed and dissolved in MeOH; the terminate concentration was ca. 1.0 mg/mL. ACFB extract was dried under vacuum, accurately weighed to about 10 mg, then dissolved in MeOH, in a 1.0 mL volumetric flask. The sample solutions were all filtered with 0.45 μm PVDF membrane filter (Millipore Sigma, Billerica, MA, USA) before use. The injection volumes of the compound and ACFB were 1 μL and 10 μL, respectively.

### 3.5. Determination of pH and Thermal Stabilities of A. cinnamomea a-Glucosidase Inhibitors

pH measurement was performed as per the methods described by Nguyen et al. (2017) [14] with slight modifications, samples were pre-treated with a large pH range of 2–11 for 30 min at 37 °C. The buffer systems (0.1 mol/L) used were glycine HCl (pH 2–4), sodium acetate (pH 5), sodium phosphate (pH 6–8) and sodium carbonate (pH 9–11). The pH of treated sample solution was adjusted to pH 7 by adding 0.25 mol/L potassium phosphate buffer (pH 7) before testing activity. The thermal stability of ACFB aGIs was also examined by pre-treating samples to a range of temperatures (40–100 °C) for 30 min, before α-glucosidase inhibitory activity was tested at 37 °C [5] using the bioassay above.

### 3.6. Statistical Analysis

The differences between the means of inhibition (%) and EC_50_ values was analyzed with the use of SAS (Statistical Analysis Software) version 9.4, provided by SAS Institute Taiwan Ltd. (Taipei, Taiwan), using Duncan’s multiple range test (α = 0.01). All tests were performed in triplicate (*n* = 3).

## 4. Conclusions

The MeOH extract of ACFB was investigated for the first time for its potent in-vitro antidiabetic effect, characterized using an α-glucosidase inhibitory activity assay. ACFB demonstrated high pH stability (pH 2–11) and thermostable properties at 40–50 °C. Five active compounds, including 25*R*-antcin K (**2**), dehydrosulphurenic acid (**3**), 25*S*-antcin B (**5**), dehydroeburicoic acid (**7**) and eburicoic acid (**8**), were successfully isolated and identified from ACFB, showing stronger α-glucosidase inhibitory effect and higher activity (EC_50_ = 0.025–0.21 mg/mL) than acarbose (EC_50_ = 0.278 mg/mL). Notably, these novel compounds were investigated for the first time for their α-glucosidase inhibitory effect in this study. The results of this study contributed to the catalogue of novel biological activities of AC, as well as its constituents. The results also suggest that ACFB is a highly rich, safe and natural source of bioactive constituents that may be developed as drugs or health foods with potential antidiabetic effects.

## Figures and Tables

**Figure 2:**
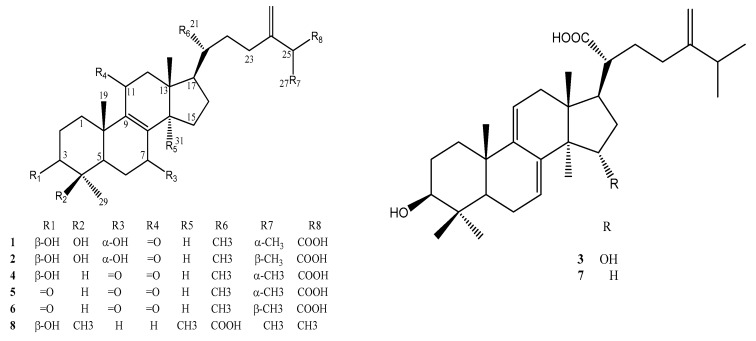
Chemical structures of purified active compounds. 25*S*-antcin K (**1**), 25*R*-antcin K (**2**), dehydrosulphurenic acid (**3**), 25*S*-antcin I (**4**), 25*S*-antcin B (**5**), 25*R*-antcin B (**6**), dehydroeburicoic acid (**7**) and eburicoic acid (**8**).

**Table 1:** α-glucosidase inhibition by recently reported natural source extracts.

Scientific Name		Solvents for Extraction	EC_50_ (mg/mL)	Ref.
**Mushroom**	**Part Used**			
Acarbose (positive control)	-		0.278 ± 0.0023	This study
*A. cinnamomea*	Fruiting body	MeOH	0.205 ± 0.0084	
*A. cinnamomea*	Mycelia	80% MeOH	310	[32]
*A. cinnamomea*	Cultural filtrate	80% MeOH	284	[32]
*Pleurotus cornucopiae*	Fruiting body	H_2_O	23.2	[16]
*Trametes pubescens*	Fruiting body	80% MeOH	≥1.0	[17]
*T. pubescens*	Fruiting body	Hot H_2_O	≥1.0	[17]
*Pleurotus eous*	Fruiting body	MeOH	0.325	[18]
*P. eous*	Fruiting body	H_2_O	0.280	[18]
*Grifola frondosa*	Fruiting body	*n*-hexane	0.0376	[19]
*Hericium erinaceum*	Fruiting body	*n*-hexane	0.0389	[19]
*Agaricus blazei*	Fruiting body	*n*-hexane	0.0528	[19]
*Ganoderma lucidum*	Fruiting body	*n*-hexane	0.0766	[19]
*Coriolus versicolor*	Fruiting body	*n*-hexane	0.125	[19]
*Phellinus linteus*	Fruiting body	*n*-hexane	0.165	[19]
**Bacteria**	**C/N Source**			
*Paenibacillus* sp.	Shrimp shells	Culture broths *	0.108	[11]
*Paenibacillus* sp.	Shrimp heads		0.455	[11]
*Paenibacillus* sp.	Crab shells		0.038	[11]
*Paenibacillus* sp.	Nutrient broths		0.081	[14]
*Paenibacillus* sp.	Squid pens		0.252	[1]
Co-culture of Bacillus mycoides and *Rhizobium* sp.	Shrimp heads		3.0	[13]
**Medicinal Plants**	**Part Used**			
Dalbergia tonkinensis	Heartwood	MeOH	0.17	[9]
*D. tonkinensis*	Bark	MeOH	0.57	[9]
*D.a tonkinensis*	Leaves	MeOH	0.78	[9]
*Terminalia bellirica*	Trunk bark	MeOH	0.41	[7]
*Terminalia corticosa*	Trunk bark	MeOH	1.42	[7]
*Cinnamomum cassia J. S. Presl.*	Trunk bark	MeOH	1.08	[6]
*Terminalia bellirica*	Leaves	MeOH	0.66	[6]
*Psidium littorale Raddi*	Leaves	MeOH	0.25	[8]

* were dehydrated to powder form, then dissolved in water before testing for α-glucosidase inhibitory activity.

**Table 2:** α-Glucosidase inhibitory activity of isolated compounds from ACFB extract.

Compd No.	Compound	EC_50_ (mg/mL)	Inhibition (%) at 0.25 mg/mL
**1**	25*S*-antcin K	≥0.25 ^ND^	37 ± 0.91 ^d^
**2**	25*R*-antcin K	0.054 ± 0.0004 ^c^	89 ± 2.03 ^b^
**3**	Dehydrosulphurenic acid	0.025 ± 0.0008 ^d^	99 ± 0.92 ^a^
**5**	25*S*-antcin B	0.21 ± 0.0076 ^b^	61 ± 2.27 ^c^
**7**	Dehydroeburicoic acid	0.018 ± 0.0002 ^d^	100 ± 1.87 ^a^
**8**	Eburicoic acid	0.012 ± 0.0004 ^d^	98 ± 2.33 ^a^
	Acarbose (positive control)	0.278 ± 0.0023 ^a^	44 ± 0.57 ^d^
	Coefficient of variation (%)	5.964010	4.021327

All inhibitors were tested within a concentration range of 0.02–0.25 μg/mL; the means of inhibitory activity, including EC_50_ (mg/mL), and inhibition (%) values with the same letters in the same column are not significantly different, based on analysis of Duncan’s multiple range test at α = 0.01, using SAS version 9.4 (SAS Institute Taiwan Ltd., Taipei, Taiwan). ND: Not detected.

## Supplementary Materials

The supplementary materials are available online.

## Abbreviations

T2D	type 2 diabetes
ACFB	*Antrodia cinnamomea* fruiting bodies
ACN	Acetone nitride
HPLC	High-performance liquid chromatography
DM	Diabetes mellitus
AGIs	α-glucosidase inhibitors
AC	*Antrodia cinnamomea*
GLUT4	Glucose transporter 4
EC_50_	Concentration of an inhibitor that inhibits 50% of enzymic activity
Ref	Reference
pNPG	*p-*nitrophenyl glucopyranoside
