# Supplementary material for: Isolation and Identification of Potent Antidiabetic Compounds from Antrodia cinnamomea—An Edible Taiwanese Mushroom
Source: Molecules. 2018 Nov 2;23(11):2864. doi: 10.3390/molecules23112864 (PMC6278467; doi:10.3390/molecules23112864)
Supplement: Supplementary file 1 [file molecules-23-02864-s001.pdf]

Supplementary materials:

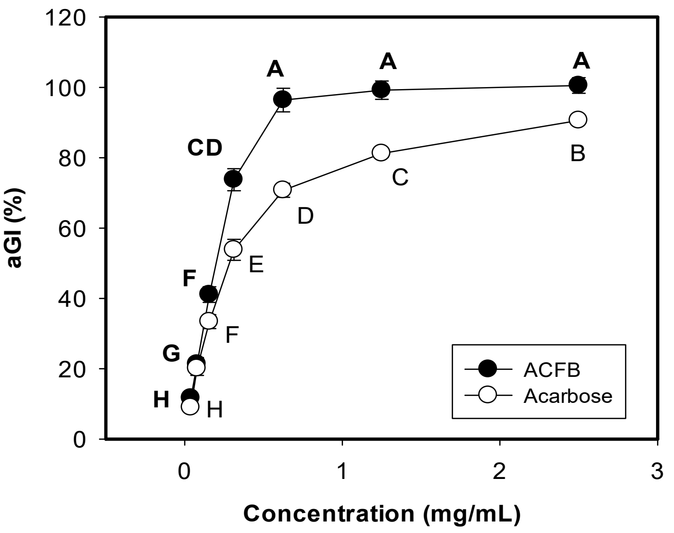

Figure S1. Comparison of α-glucosidase inhibitory activity, aGI (%) of ACFB extract and acarbose. The means of α-glucosidase inhibitory activity (aGI%) values with the same letter are not significantly different, based on Duncan's multiple range test (alpha = 0.01), using Statistical Analysis Software (SAS) version 9.4. CV: coefficient of variation.

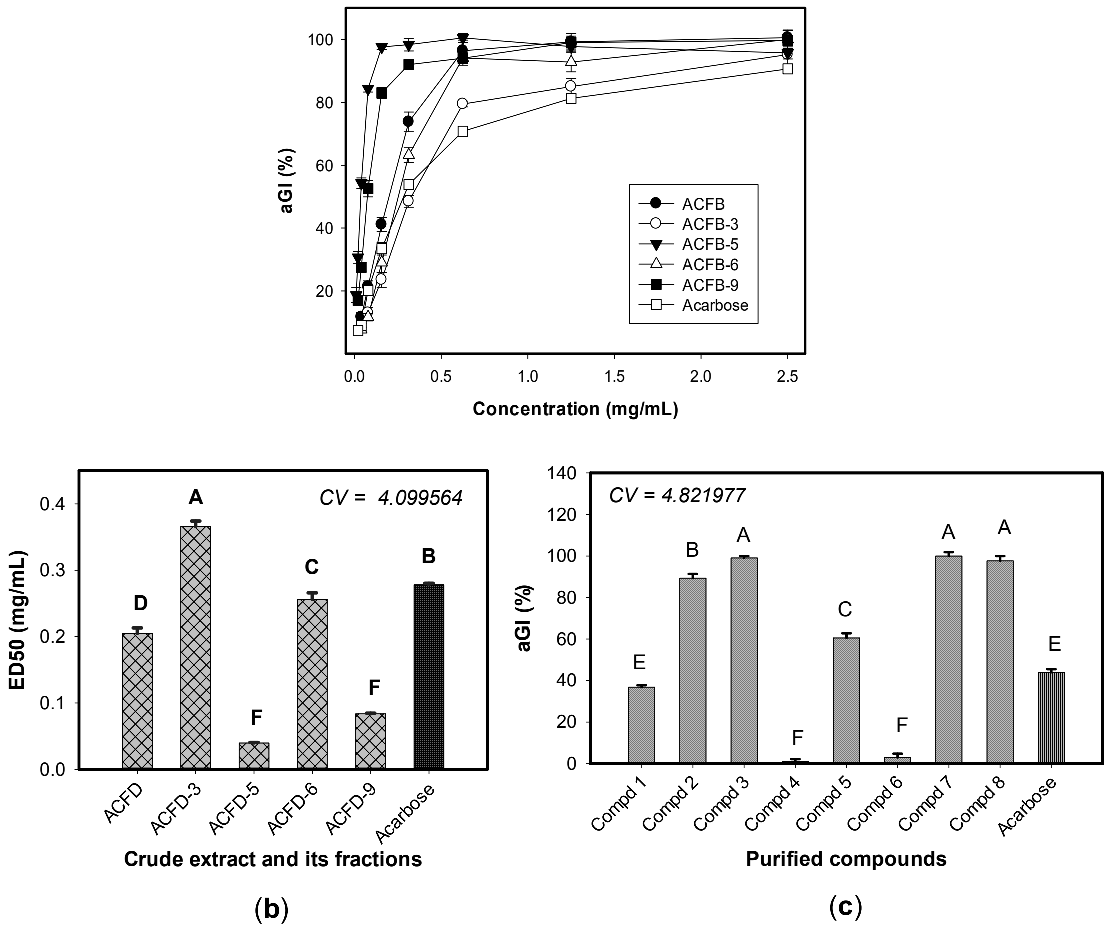

Figure S2. α-Glucosidase inhibitory activity of ACFB extract, its fractions and acarbose, expressed as % (**a**) and EC_{50} (**b**), and the α-glucosidase inhibition of purified compounds (**c**). The means of EC_{50} (**b**) and % (**c**) values with the same letter are not significantly different, based on Duncan's multiple range test (alpha = 0.01) using Statistical Analysis Software (SAS) version 9.4. CV: coefficient of variation.

Table S1. ^{13}C-NMR spectroscopic data of compounds **1~8** (C_5D_5N, δ in ppm, J in Hz).

No.	**1**	**2**	**3**	**4**	**5**	**6**	**7**	**8**
	δ_H (J in Hz)	δ_H (J in Hz)	δ_H (J in Hz)	δ_H (J in Hz)	δ_H (J in Hz)	δ_H (J in Hz)	δ_H (J in Hz)	δ_H (J in Hz)
1	3.050 (d, 12.8)	3.035 (d, 12.8)	2.694 (m)	2.679 (m)	3.117 (m)	3.139 (m)	2.646 (m)	2.619 (m)
	2.050 (m)	2.043 (m)	1.895 (m)	1.828 (m)	1.478 (m)	1.428 (m)	1.900 (m)	1.916 (m)
2	2.719 (m)	2.704 (m)	2.614 (m)	1.799 (m)	2.526 (m)	2.525 (m)	2.535 (m)	2.439 (m)
	1.907 (m)	1.895 (m)	1.949 (m)	1.284 (m)	2.387 (m)	2.386 (m)	1.954 (m)	1.966 (m)
3	4.026 (s)	4.014 (s)	3.422 (t, 8.0)	3.852 (s)			3.432 (t, 8.0)	3.406 (t, 7.2)
4				1.664 (m)	2.419 (m)	2.420 (m)		
5	2.096 (m)	2.116 (m)	2.587 (m)	2.589 (m)	1.837 (m)	1.829 (m)	2.619 (m)	2.592 (m)
6	2.394 (m)	2.392 (m)	2.350 (m)	2.633 (m)	2.507 (m)	2.510 (m)	2.347 (m)	2.381 (m)
	2.679 (m)	2.669 (m)	2.188 (m)	2.416 (m)	2.467 (m)	2.489 (m)	2.127 (m)	2.070 (m)
7	4.583 (t, 8.4)	4.578 (t, 8.4)	6.477 (d, 4.8)				5.604 (d, 4.0)	2.023 (m)
								1.810 (m)
11			overlapping				5.361 (d, 4.8)	2.229 (m)
								1.976 (m)
12	2.928 (d, 13.6)	2.919 (d, 13.6)	2.649 (m)	2.911 (d, 13.2)	2.945 (d, 13.6)	2.990 (d, 13.6)	2.549 (m)	2.600 (m)
	2.427 (m)	2.425 (m)	2.378 (m)	2.445 (m)	2.433 (m)	2.454 (m)	2.320 (m)	2.244 (m)
14	2.615 (m)	2.620 (m)		2.735 (m)	2.691 (m)	2.715 (m)		
15	2.648 (m)	2.636 (m)	4.764 (dd, 6.0)	2.730 (m)	2.670 (m)	2.700 (m)	2.507 (m)	2.416 (m)
	2.130 (m)	2.120 (m)		1.516 (m)	1.220 (m)	1.272 (m)	1.512 (m)	1.527 (m)
16	1.879 (m)	1.861 (m)	1.843 (m)	1.828 (m)	1.863 (m)	1.860 (m)	1.800 (m)	1.746 (m)
	1.285 (m)	1.280 (m)	1.280 (m)	1.234 (m)	1.237 (m)	1.242 (m)	1.261 (m)	1.269 (m)
17	1.388 (m)	1.385 (m)	1.450 (m)	1.364 (m)	1.365 (m)	1.396 (m)	1.444 (m)	1.464 (m)
18	0.862 (s)	0.851 (s)	1.049 (s)	0.651 (s)	0.646 (s)	0.677 (s)	1.114 (s)	1.048 (s)
19	2.011 (s)	1.994 (s)	1.077 (s)	1.411 (s)	1.554 (s)	1.578 (s)	1.200 (s)	1.220 (s)
20	1.367 (m)	1.368 (m)	2.336 (m)	1.342 (m)	1.345 (m)	1.377 (m)	2.364 (m)	2.369 (m)
21	0.862 (s)	0.851 (s)		0.829 (d, 5.2)	0.851 (d, 5.2)	0.874 (d, 5.2)		
22	1.712 (m)	1.738 (m)	1.914 (m)	1.637 (m)	1.642 (m)	1.707 (m)	1.776 (m)	1.717 (m)
	1.265 (m)	1.251 (m)	1.297 (m)	1.213 (m)	1.254 (m)	1.272 (m)	1.277 (m)	1.245 (m)
23	2.463 (m)	2.449 (m)	2.222 (m)	2.346 (m)	2.404 (m)	2.436 (m)	2.250 (m)	2.261 (m)
	2.179 (m)	2.180 (m)	2.156 (m)	2.135 (m)	2.150 (m)	2.176 (m)	2.164 (m)	2.009 (m)
25	3.409 (brq)	3.418 (brq)	2.206 (m)	3.401 (q, 6.4)	3.399 (brq, 7.2)	3.455 (brq, 7.2)	2.300 (m)	2.293 (m)

26			0.969 (d, 3.6)				0.997 (s)	1.007 (s)
27	1.465 (d, 7.2)	1.453 (d, 7.2)	0.952 (d, 3.2)	1.453 (d, 7.2)	1.457 (d, 6.8)	1.495 (d, 6.8)	0.997 (s)	1.007 (s)
28	5.182 (s)	5.186 (s)	4.851 (s)	5.172 (s)	5.173 (s)	5.235 (s)	4.923 (m)	4.908 (s)
	5.035 (s)	5.024 (s)	4.819 (s)	5.020 (s)	5.020 (s)	5.061 (s)	4.877 (m)	4.868 (s)
29	1.694 (s)	1.683 (s)	1.413 (s)	1.022 (d)	0.982 (d, 6.4)	1.012 (d, 6.4)	1.015 (t, 2.8)	0.996 (s)
30			1.142 (s)				1.056 (s)	1.059 (s)
31			1.099 (s)				10.56 (s)	1.023 (s)

Table S2. ^{13}C-NMR spectroscopic data of compounds **1~8** (C_5D_5N, δ in ppm).

Carbon	1	2	3	4	5	6	7	8
1	29.64	29.62	36.66	28.39	34.90	34.94	36.32	36.10
2	26.71	26.70	28.67	29.93	37.73	37.77	28.65	26.81
3	74.65	74.64	77.91	69.10	209.86	209.88	77.99	77.98
4	73.92	73.91	39.39	35.09	43.88	43.92	39.32	37.37
5	43.45	43.44	49.53	41.44	48.87	48.90	49.71	50.88
6	30.12	30.11	23.37	38.34	39.16	39.20	23.53	21.24
7	70.75	70.75	122.18	202.21	200.72	200.77	121.31	27.46
8	154.22	154.21	141.77	144.68	145.45	145.49	142.77	135.16
9	143.90	143.90	146.86	153.72	151.91	151.95	146.61	134.27
10	38.70	38.68	37.78	39.11	38.58	38.62	37.83	39.50
11	201.46	201.45	116.10	202.79	202.62	202.67	116.58	18.70
12	58.76	58.76	36.26	57.43	57.41	57.46	35.98	30.86
13	47.68	47.88	44.76	47.19	47.18	47.23	44.25	49.85
14	53.72	53.72	52.33	49.34	49.41	49.46	50.47	44.89
15	25.41	25.42	73.61	25.18	25.24	25.29	31.58	32.71
16	28.22	28.17	39.17	27.84	27.95	27.96	27.25	28.67
17	54.76	54.82	46.29	53.79	53.90	53.99	48.11	49.12
18	12.43	12.43	16.68	11.89	12.03	12.08	16.24	19.40
19	20.90	20.89	22.93	16.08	16.21	16.24	22.96	16.32
20	36.15	36.18	48.74	35.69	35.80	35.88	49.07	47.69
21	18.56	18.60	178.62	18.33	18.48	18.56	178.50	178.55
22	34.36	34.45	32.55	34.06	34.20	34.34	31.75	29.34
23	31.84	31.68	31.69	31.59	31.71	31.57	32.72	31.78
24	150.28	150.42	155.60	150.01	150.21	150.34	155.84	155.85
25	46.46	46.70	34.01	46.31	46.46	46.74	34.20	34.19

26	176.83	176.85	21.72	176.71	176.77	176.82	21.87	21.88
27	16.94	17.11	21.85	16.79	16.95	17.16	21.99	22.00
28	110.42	110.36	106.97	110.34	110.50	110.52	107.04	107.01
29	27.98	27.97	18.15	16.27	11.52	11.55	16.60	16.32
30			28.50				25.85	24.49
31			16.50				28.80	28.61

^{1}H NMR and ^{13}C-NMR spectrums, and HREIMS of 6 active α-glucosidase inhibitors isolated from methanolic extract of *Antrodia cinnamomea* fruiting bodies (ACFD).

1. **^{1}H-NMR and ^{13}C-NMR spectrums, and HREIMS of 25*S*-antcinK (1)**

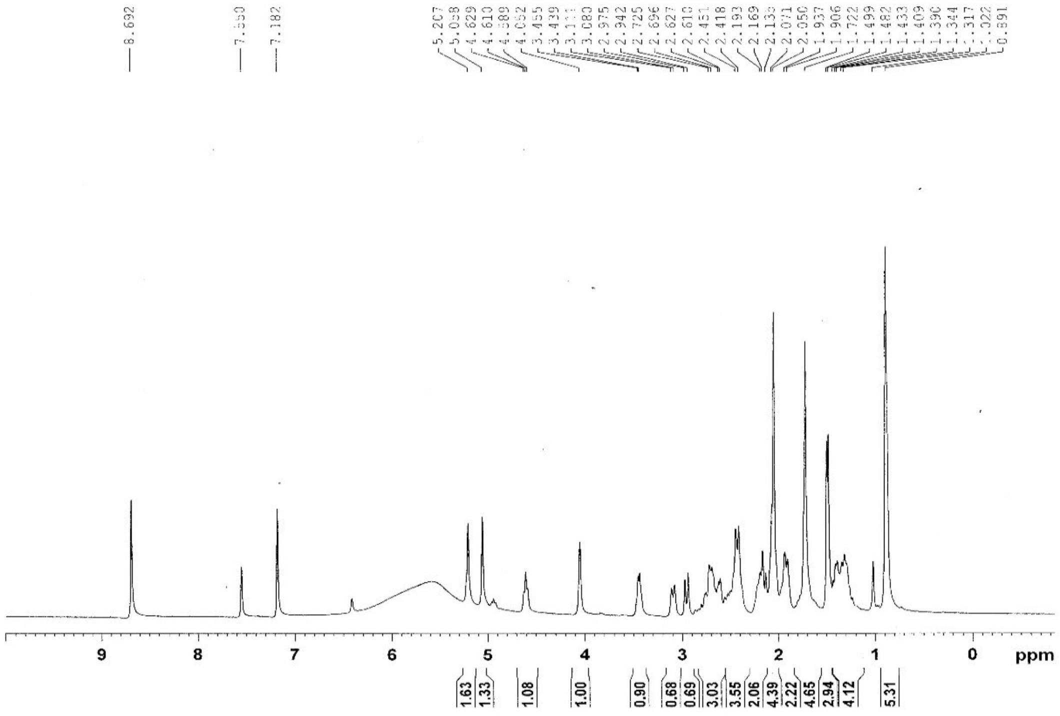

Figure S3. ^{1}H NMR spectrum of 25*S*-antcinK (**1**) measured in 400 MHz in pyridine-d_5.

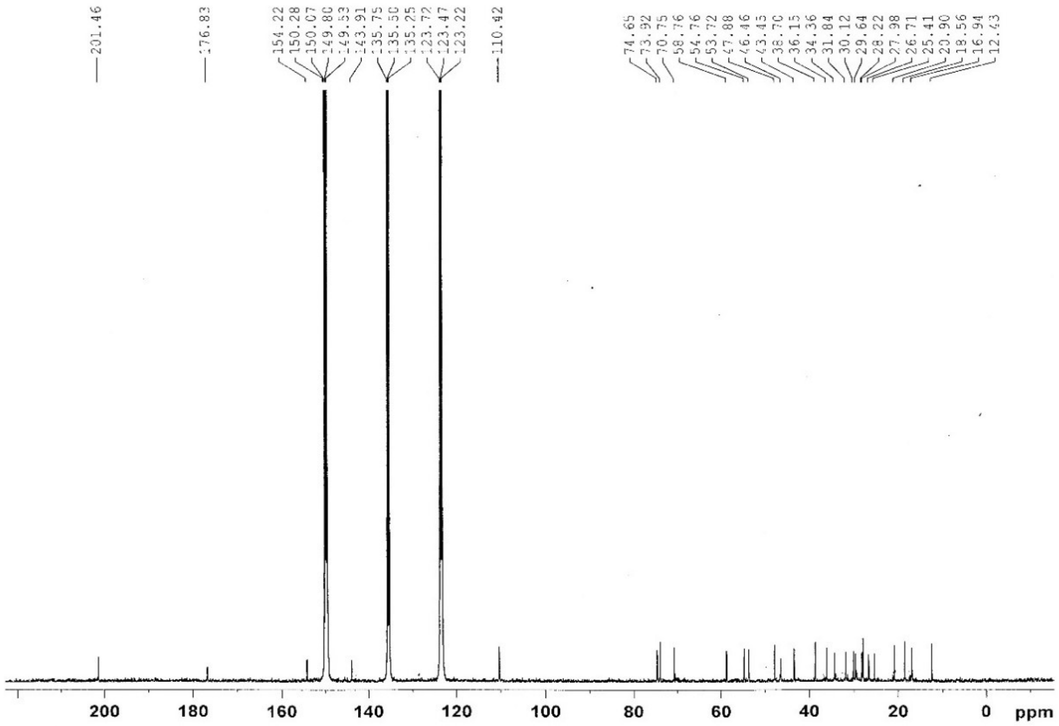

Figure S4. ^{13}C-NMR spectrum of 25*S*-antcinK (**1**) measured in 100 MHz in pyridine-d_5.

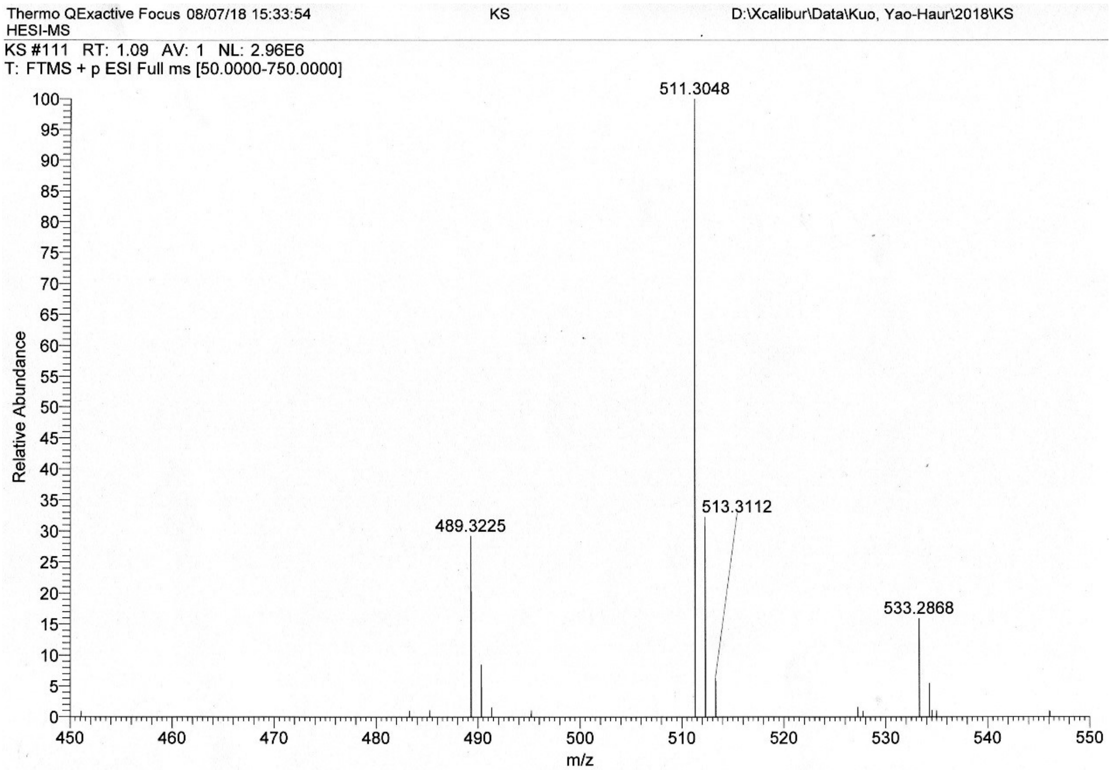

Figure S5. The HREIMS of 25*S*-antcinK (**1**), M=488, [M+Na]$^+$

2. ^{1}H-NMR and ^{13}C-NMR spectrums, and HREIMS of 25*R*-antcin K (2)

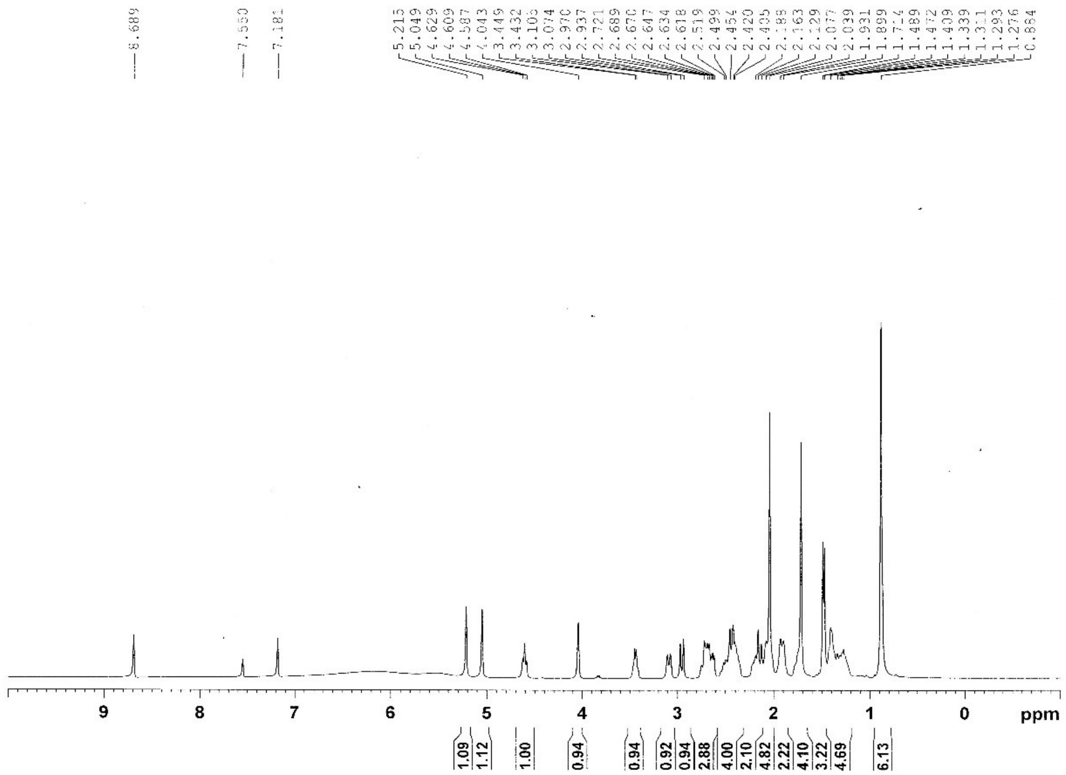

Figure S6. ^{1}H NMR spectrum of 25*R*-antcin K (**2**) measured in 400 MHz in pyridine-d_5.

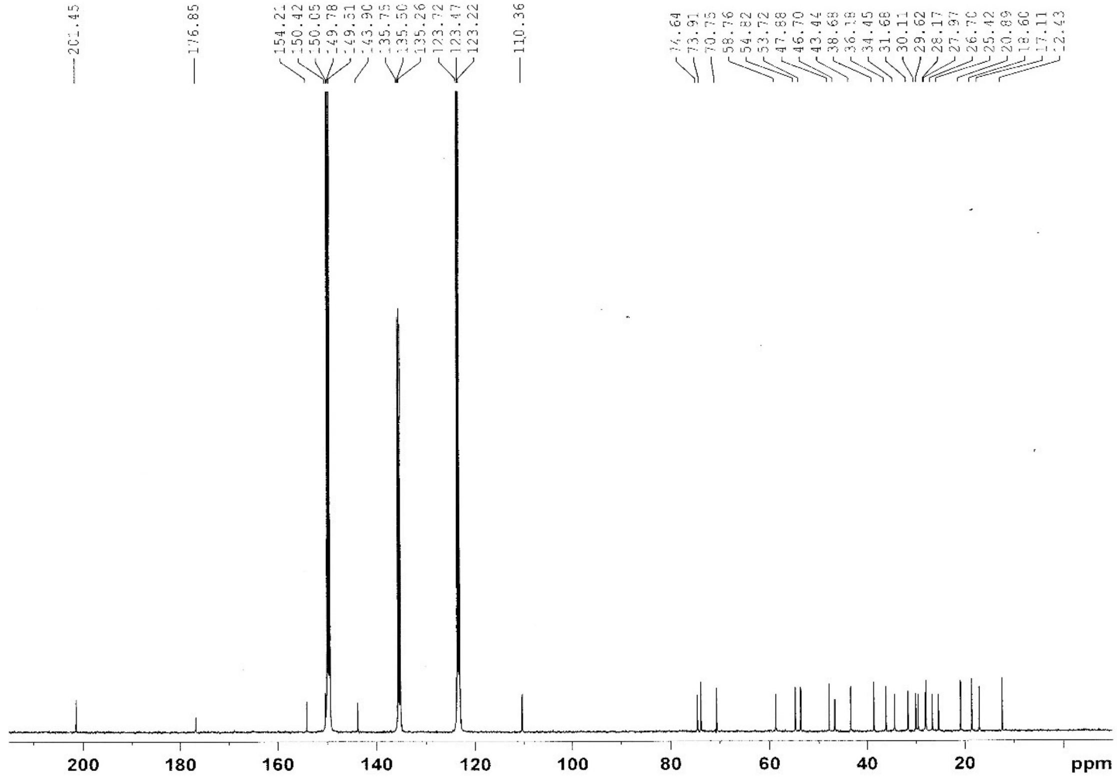

Figure S7. ^{13}C-NMR spectrum of 25*R*-antcin K (**2**) measured in 100 MHz in pyridine-*d*$_5$.

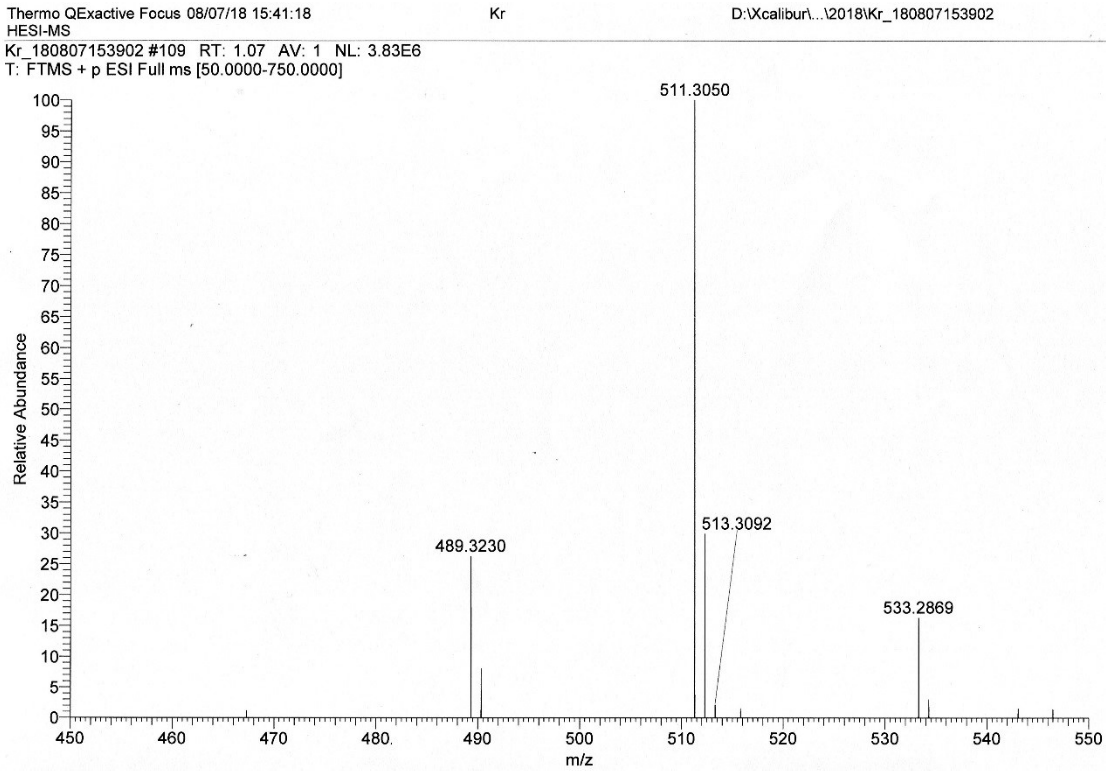

Figure S8. The HREIMS of25*R*-antcin K (**2**), M=488, [M+Na]$^+$

3. ^{1}H NMR and ^{13}C-NMR spectrums, and HREIMS of dehydrosulphurenic acid (3)

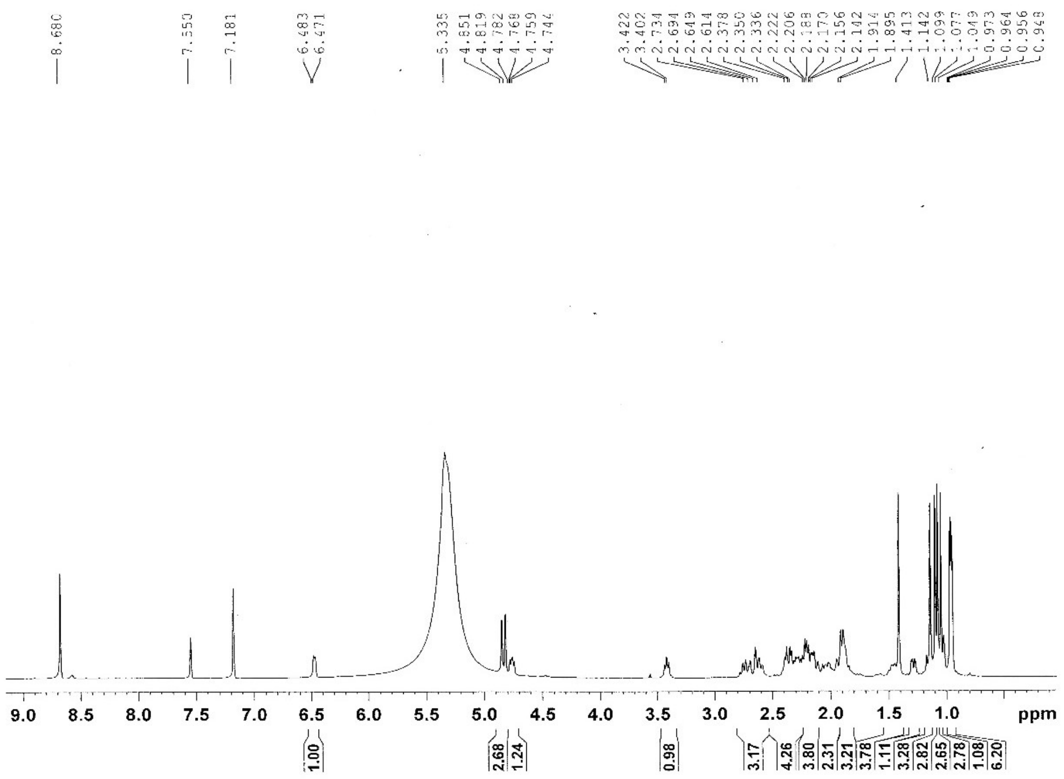

Figure S9. ^{1}H NMR spectrum of dehydrosulphurenic acid (**3**) measured in 400 MHz in pyridine-d_5.

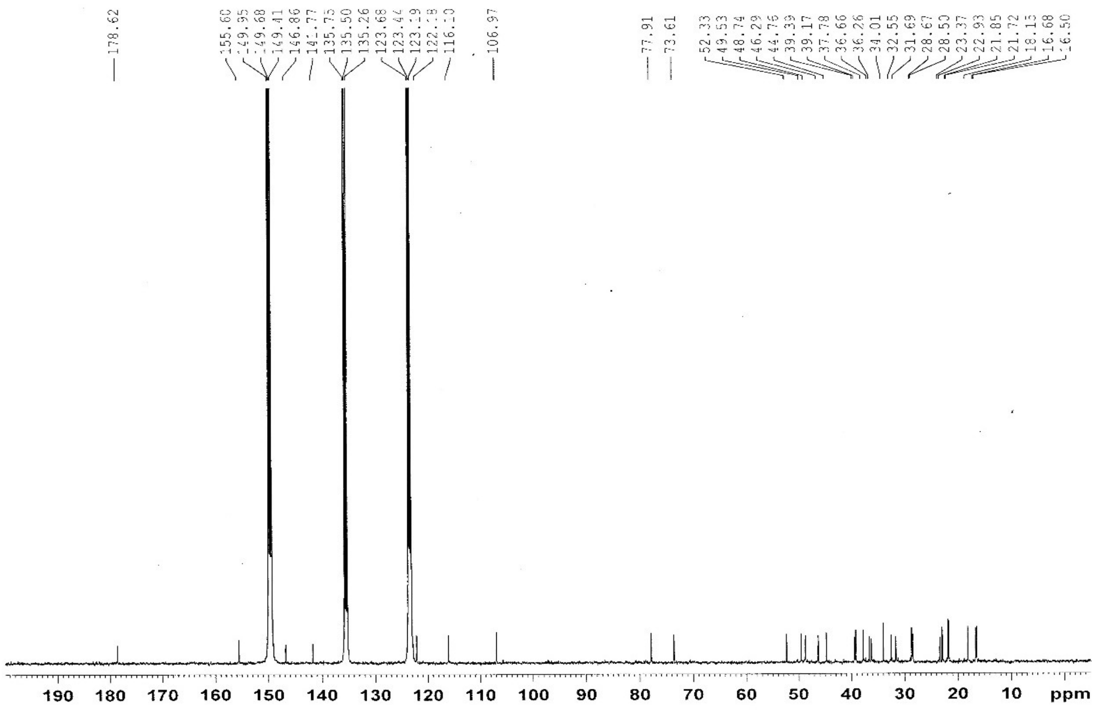

Figure S10. ^{13}C-NMR spectrum of dehydrosulphurenic acid (**3**) measured in 100 MHz in pyridine-d_5.

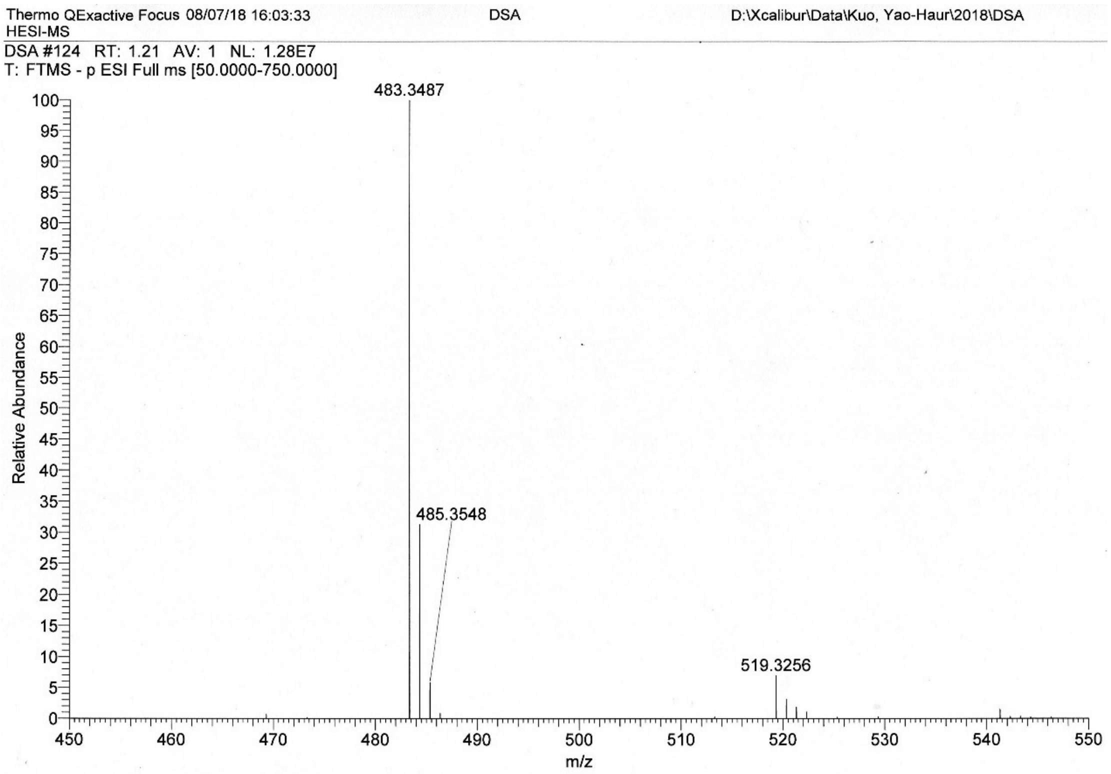

Figure S11. The HREIMS of dehydrosulphurenic acid (**3**), M=484, [M-H]⁻

4. ^{1}H NMR and ^{13}C-NMR spectrums, and HREIMS of 25*S*-antcin I (**4**)

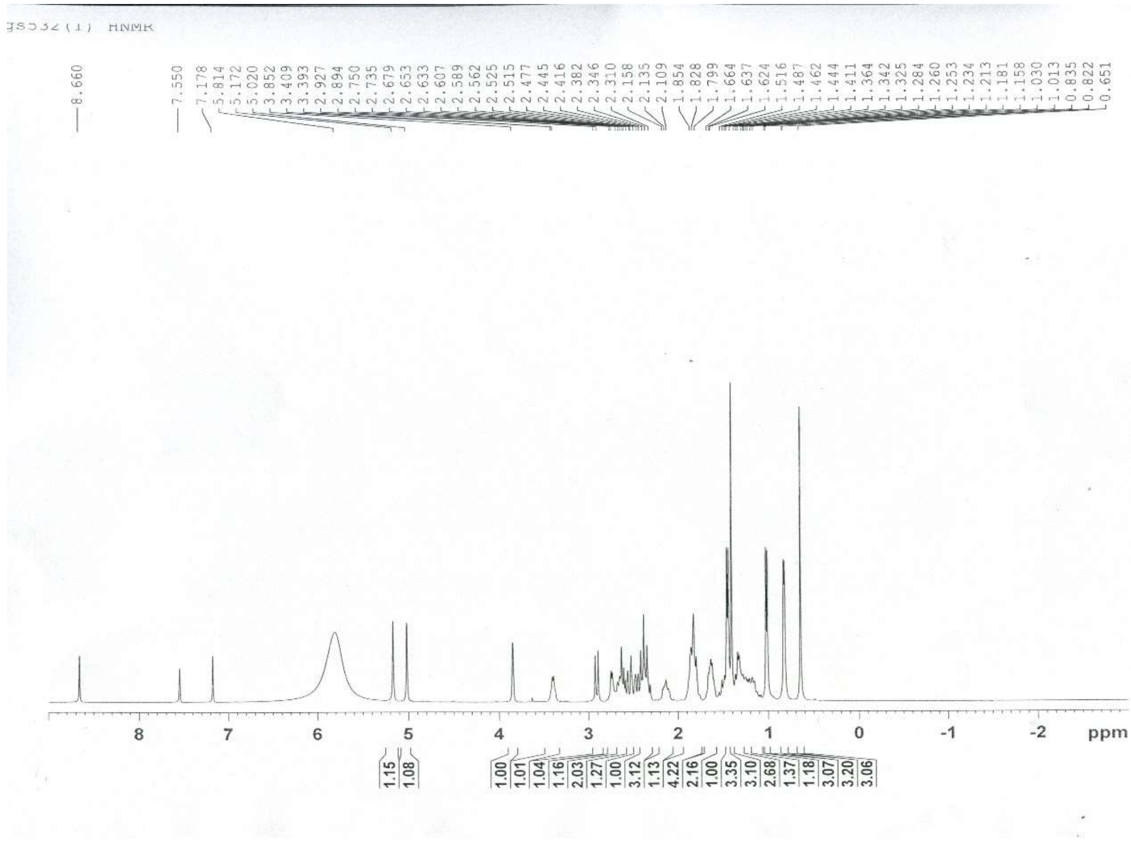

Figure S12. ^{1}H NMR spectrum of 25*S*-antcin I (**4**) measured in …………………………...

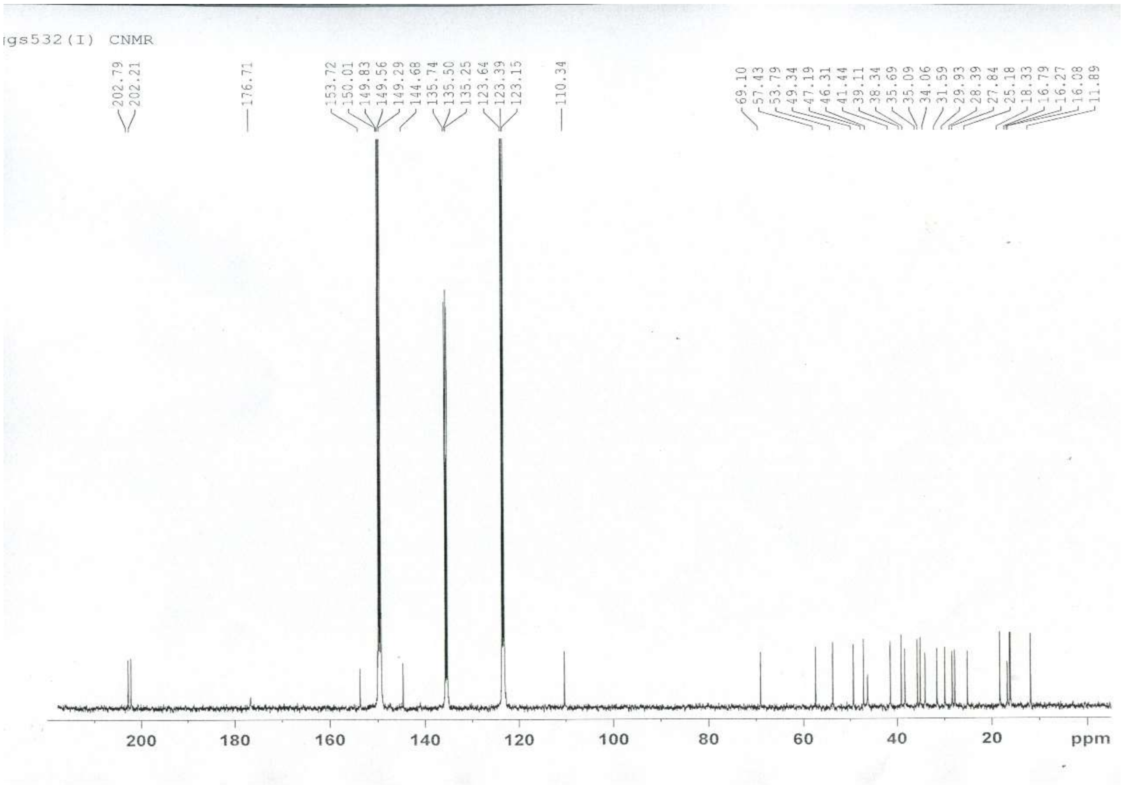

Figure S13. ^{13}C-NMR spectrum of 25*S*-antcin I (**4**) measured in ……………………..

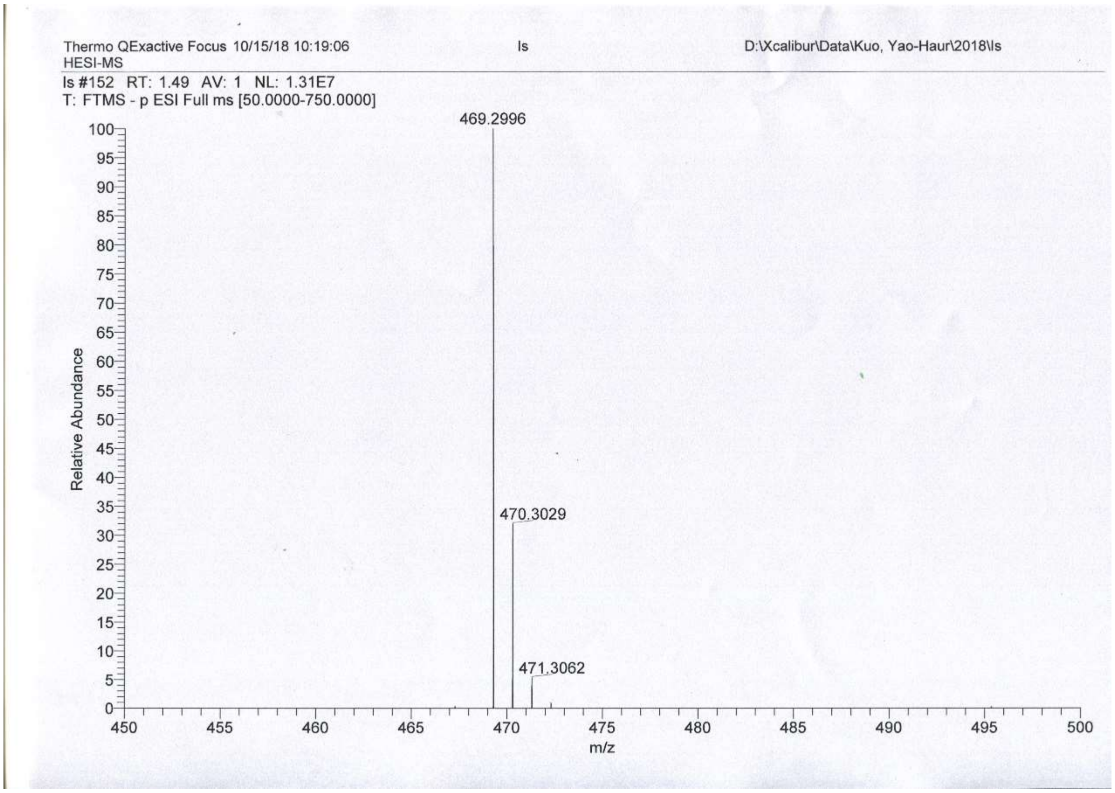

Figure S14. The HREIMS of 25*S*-antcin I (**4**), M=470, [M-H]$^-$

5. ^{1}H NMR and ^{13}C-NMR spectrums, and HREIMS of 25*S*-antcin B (5)

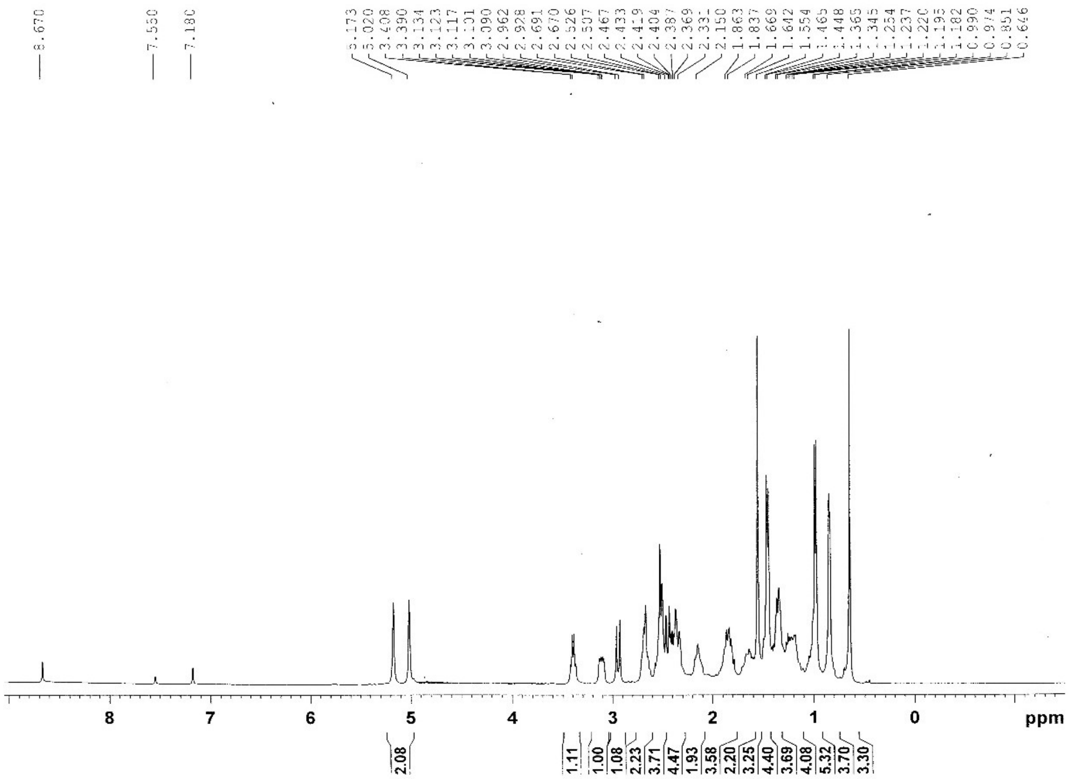

Figure S15. ^{1}H NMR spectrum of 25*S*-antcin B (**5**) measured in 400 MHz in pyridine-d_5.

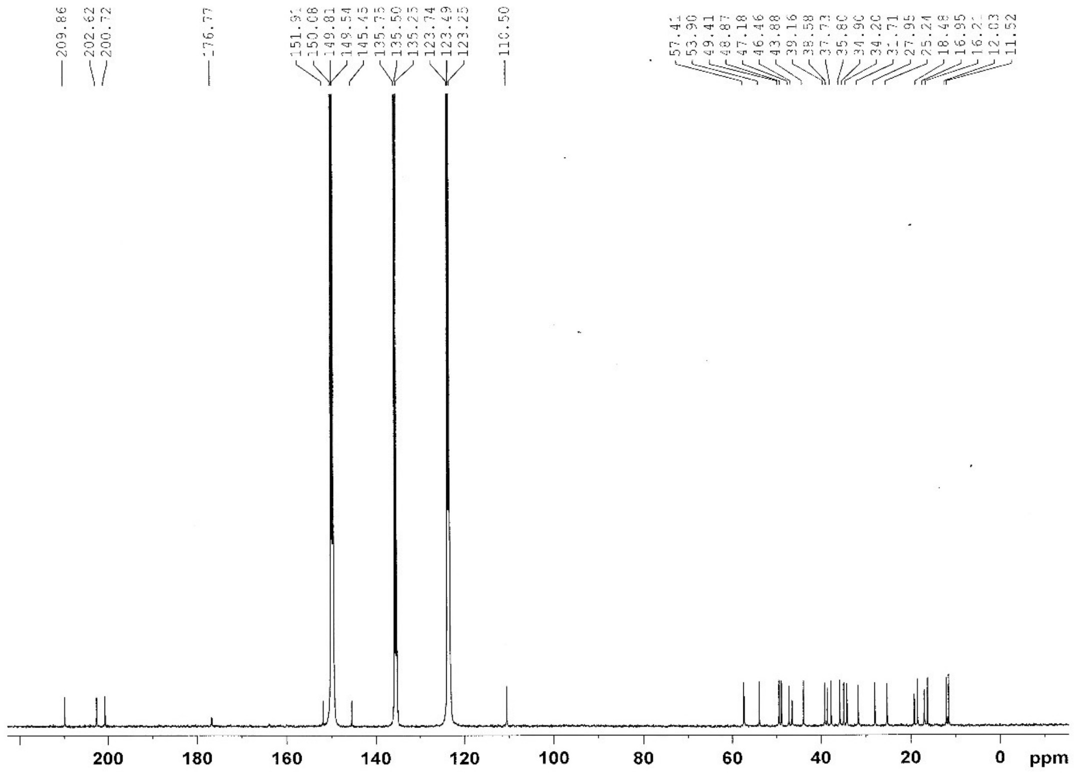

Figure S16. ^{13}C-NMR spectrum of 25*S*-antcin B (**5**) measured in 100 MHz in pyridine-d_5.

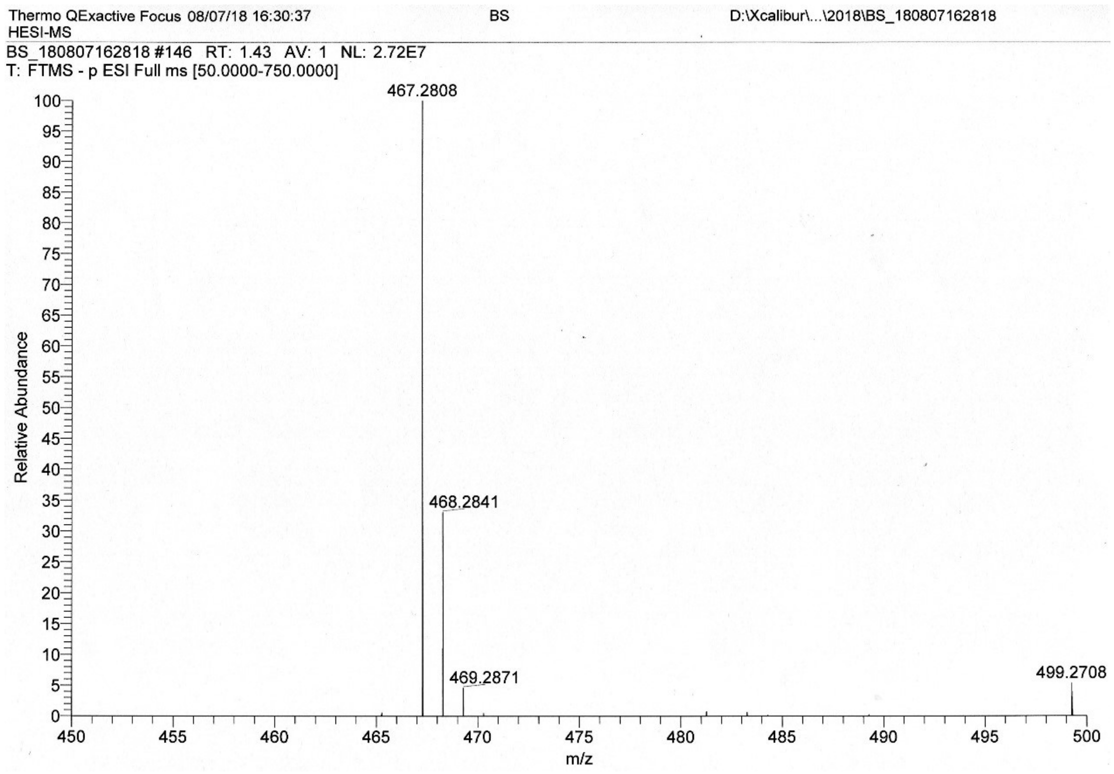

Figure S17. The HREIMS of 25*S*-antcin B (**5**), M=468, [M-H]$^-$

6. ^{1}H NMR and ^{13}C-NMR spectrums, and HREIMS of 25*R*-antcin B (**6**)

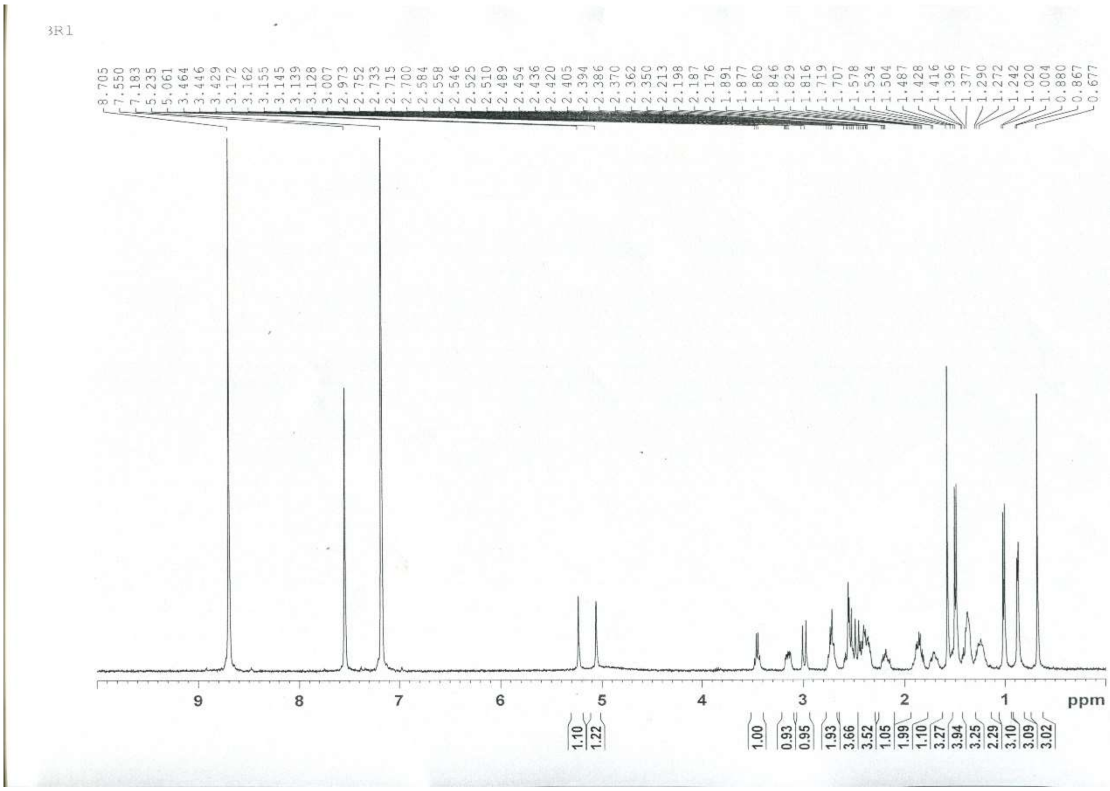

Figure S18. ^{1}H NMR spectrum of 25*R*-antcin B (**6**) measured in 400 MHz in pyridine-d_5.

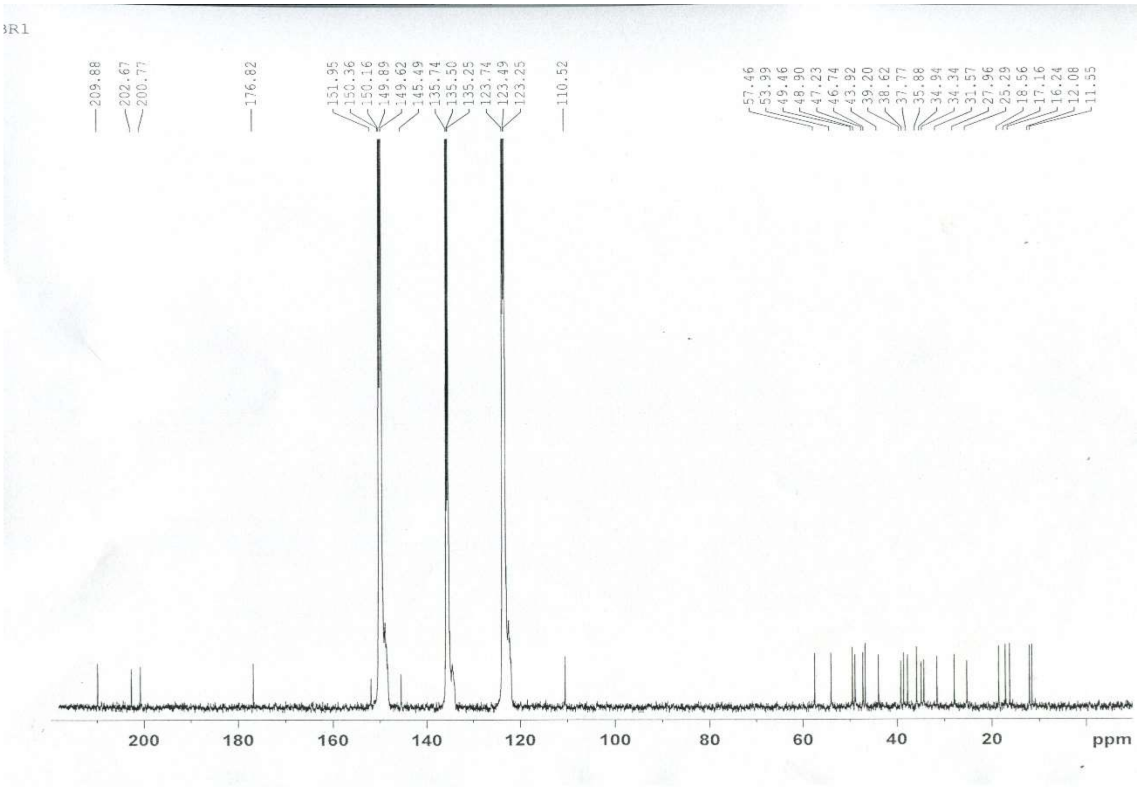

Figure S19. ^{13}C-NMR spectrum of 25*R*-antcin B (**6**) measured in 100 MHz in pyridine-d_5.

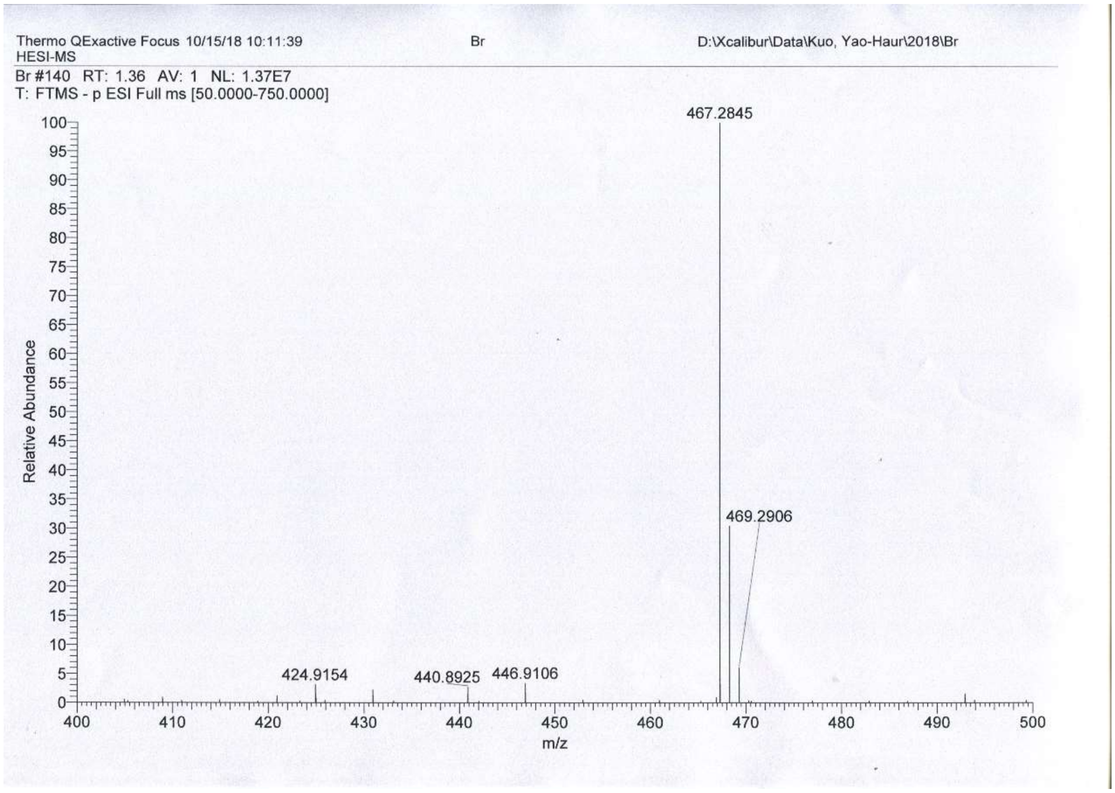

Figure S20. The HREIMS of 25*R*-antcin B (**6**), M=468, [M-H]$^-$

7. ¹H NMR and ¹³C-NMR spectrums, and HREIMS of dehydroeburicoic acid (7)

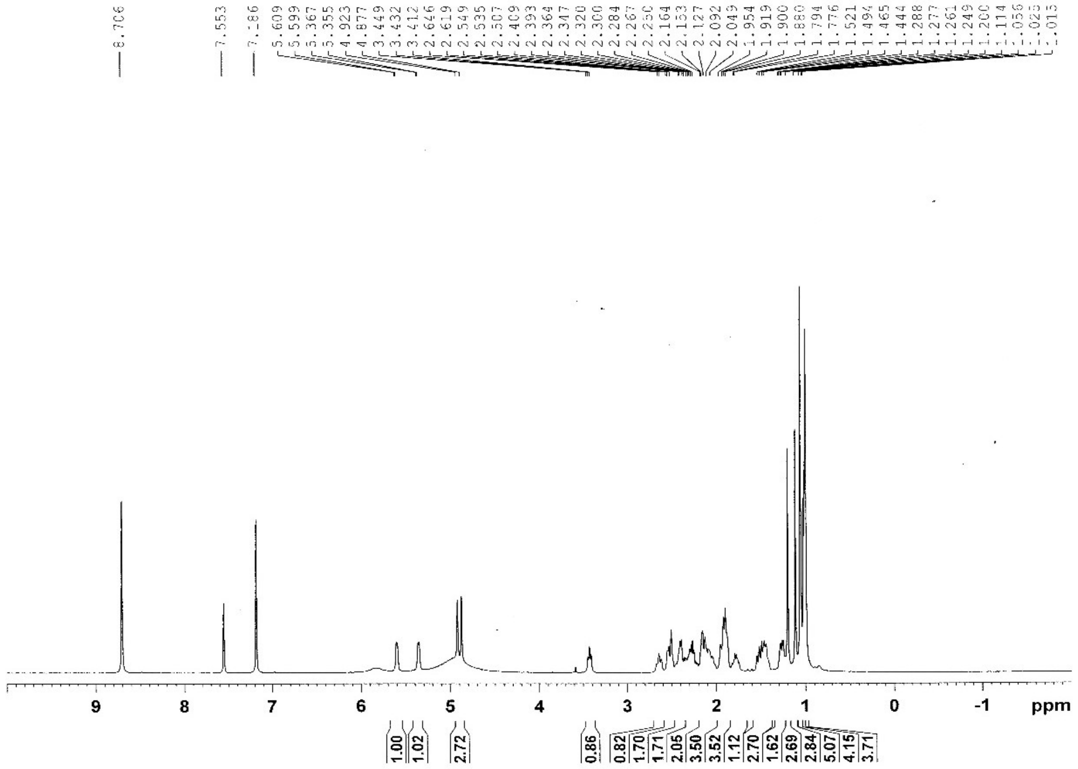

Figure S21. ¹H NMR spectrum of dehydroeburicoic acid (**7**) measured in 400 MHz in pyridine-d_5.

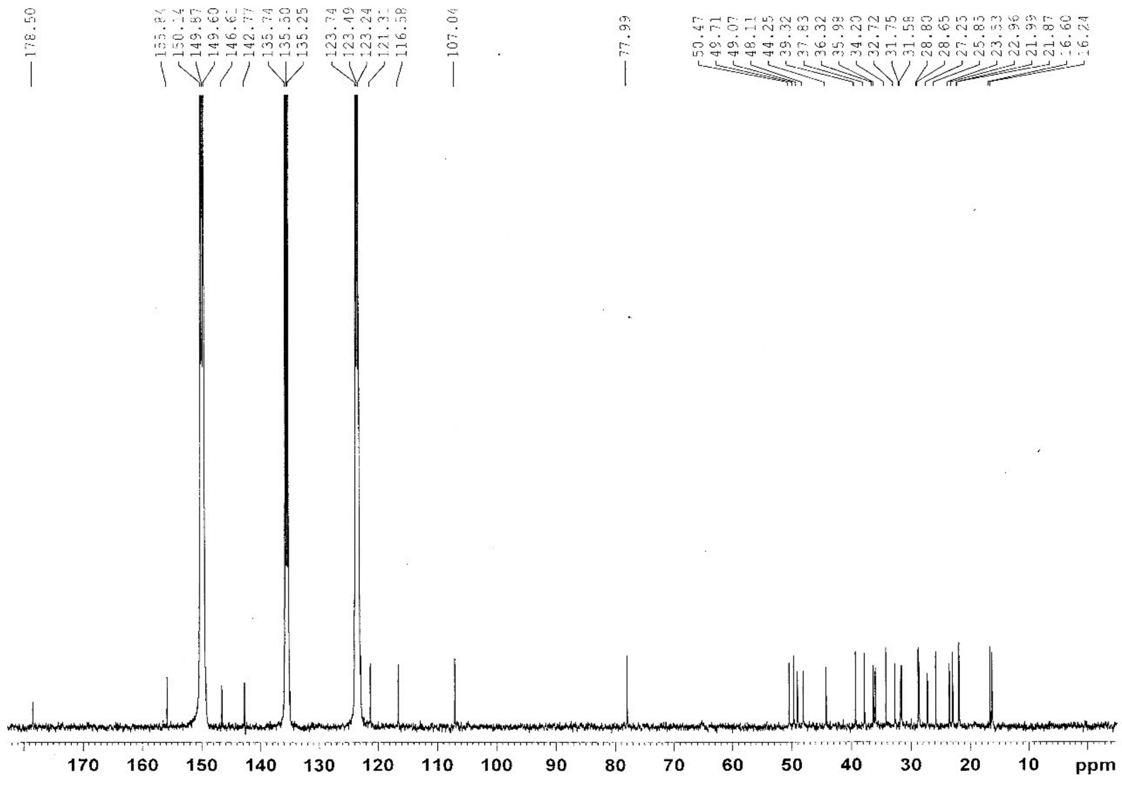

Figure S22. ¹³C-NMR spectrum of dehydroeburicoic acid (**7**) measured in 100 MHz in pyridine-d_5.

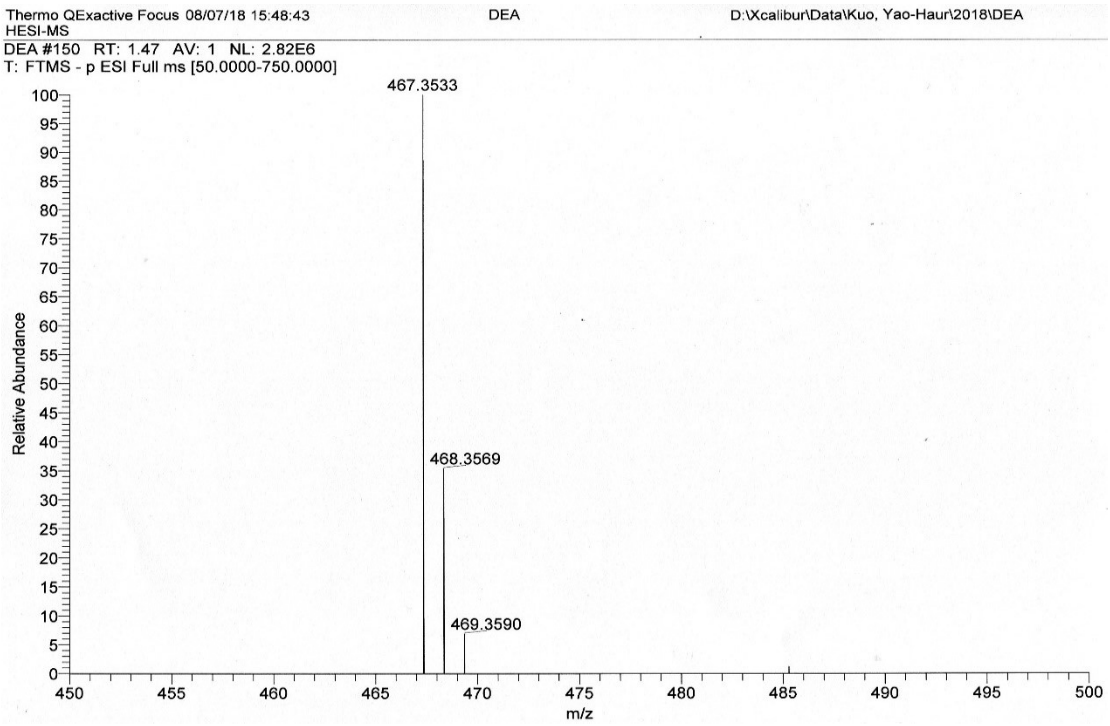

Figure S23. The HREIMS of dehydroeburicoic acid (**7**), M=468, [M-H]-

8. ^{1}H NMR and ^{13}C-NMR spectrums, and HREIMS of eburicoic acid (8)

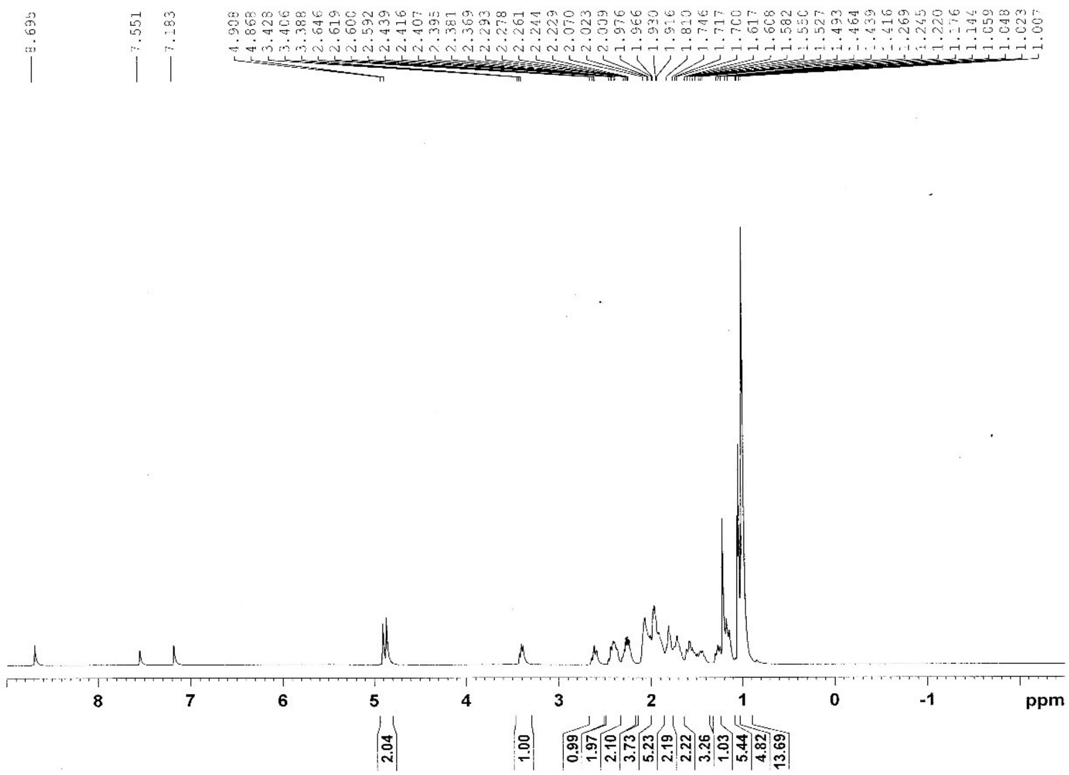

Figure S24. ^{1}H NMR spectrum of eburicoic acid (**8**) measured in 400 MHz in pyridine-d_5.

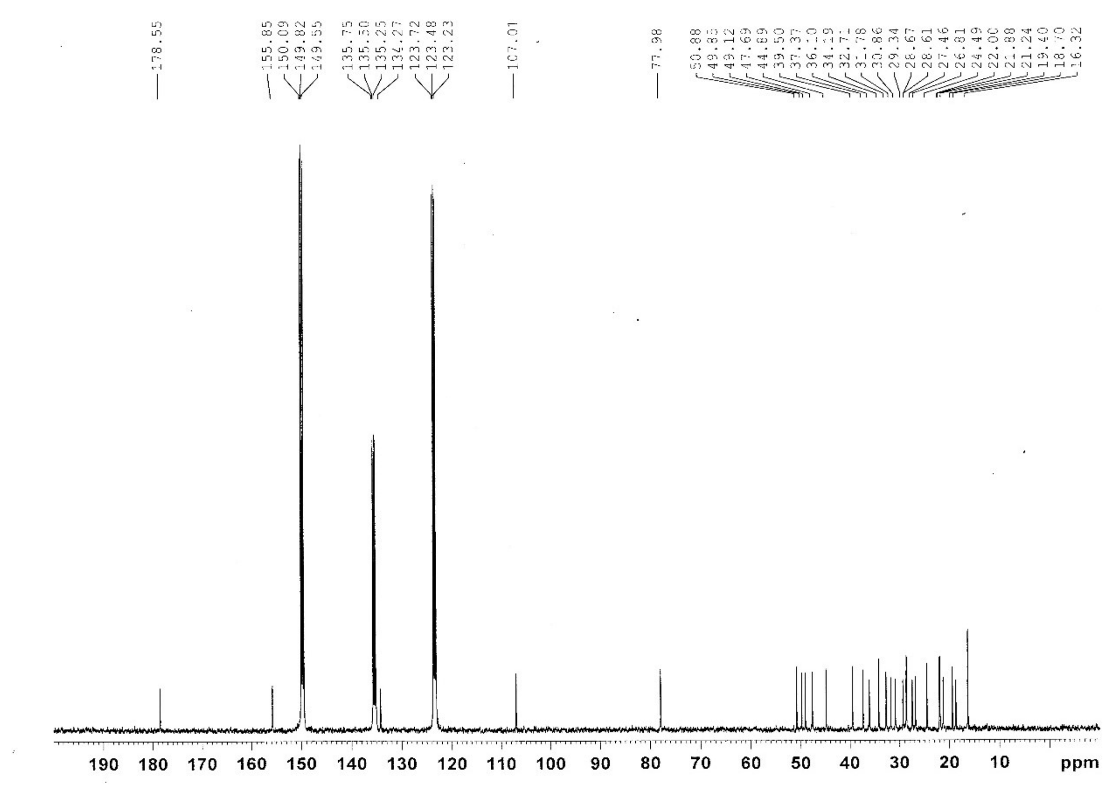

Figure S25. ^{13}C-NMR spectrum of eburicoic acid (**8**) measured in 100 MHz in pyridine-d_5.

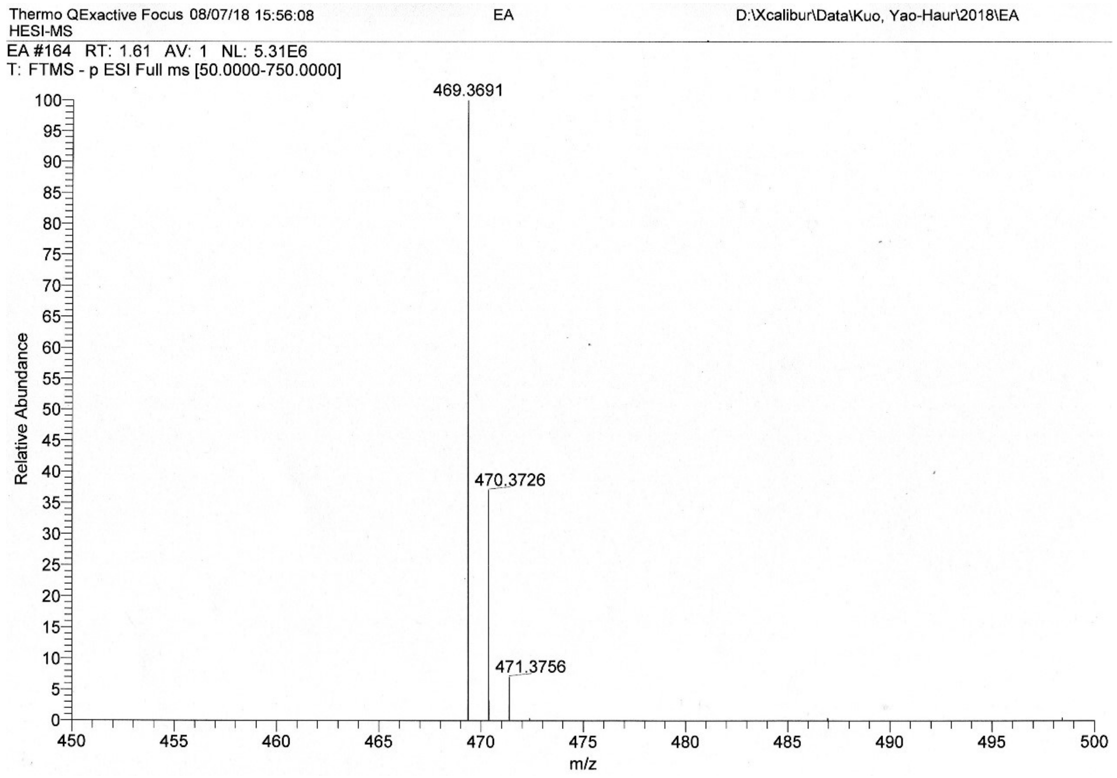

Figure S26. The HREIMS of eburicoic acid (**8**), M=470, [M-H]$^-$
